# Transdisciplinary fetal-neonatal neurology training integrates women’s and children’s health with life-course brain capital strategies: a narrative review

**DOI:** 10.3389/fneur.2026.1756627

**Published:** 2026-05-25

**Authors:** Mark S. Scher, Shazia Adalat, Harris Eyre, Michael E. Msall, Sharon L. Ramey, Craig T. Ramey, Ana Cristancho, Anastasiya Markvarde

**Affiliations:** 1 Departments of Pediatrics and Neurology, Case Western Reserve University School of Medicine, Cleveland, OH, United States; 2 Evelina London’s Children’s Hospital, Guy’s and St Thomas’ NHS Foundation Trust, King’s College London, London, United Kingdom; 3 Brain Capital Alliance, San Francisco, CA, United States; 4 Center for Health and Biosciences, The Baker Institute for Public Policy, Rice University, Houston, TX, United States; 5 Meadows Mental Health Policy Institute, Dallas, TX, United States; 6 Institute for Mental and Physical Health and Clinical Translation (IMPACT), Deakin University and Barwon Health, Geelong, VIC, Australia; 7 Department of Psychiatry and Behavioral Sciences, Baylor College of Medicine, Houston, TX, United States; 8 Department of Pediatrics, Section of Developmental and Behavioral Pediatrics, University of Chicago School of Medicine, Chicago, IL, United States; 9 Fralin Biomedical Research Institute (FBRI), Roanoke, VA, United States; 10 Virginia Tech (VT), Blacksburg, VA, United States; 11 Virginia Tech Carilion School of Medicine (VTSOM), Roanoke, VA, United States; 12 FBRI Neuromotor Research Clinic, Roanoke, VA, United States; 13 Fralin Biomedical Research Institute at VTC, Roanoke, VA, United States; 14 Departments of Psychology and Neuroscience, Blacksburg, VA, United States; 15 Department of Human Development, College of Liberal Arts and Human Science, Virginia Tech, Blacksburg, VA, United States; 16 Department of Pediatrics, School of Medicine, Blacksburg, VA, United States; 17 Division of Child Neurology, Children’s Hospital of Philadelphia, Philadelphia, PA, United States; 18 Departments of Pediatrics and Neurology, Perelman School of Medicine at the University of Pennsylvania, Philadelphia, PA, United States; 19 Independent Women’s Health and Innovation Expert, Espoo, Finland

**Keywords:** cultural neuroscience, fetal-neonatal neurology, integrative woman’s-children’s health, neural exposome, neurologic-mental health disorders, neuroplasticity, transdisciplinary care

## Abstract

Neurological and mental health disorders affect over one-third of the global population. Healthcare systems continue to treat maternal brain health and neurodevelopment as separate domains. Critical intervention windows continue to be missed before and during the first 1,000 days after conception. Current fetal-neonatal neurology training reflects healthcare fragmentation. Specialty-siloed education impedes integrative critical thinking that more successfully capitalizes on pre-conception and gestational neuroprotective opportunities. This narrative review presents perspectives that argue for a transdisciplinary approach among stakeholders that advances life-course brain healthcare. Integrative women’s and children’s health, the developmental origins of health and disease, cultural neuroscience, and brain health capital frameworks collectively contribute to an educational, practice and research model. This methodology more productively addresses public health priorities to offer equitable global brain health care based on knowledge of intersectionality. We propose that every pregnancy represents a brain health intervention opportunity. Healthcare bundles have been defined as a set of three to five evidence-based interventions to assess the quality and outcome of medical care choices. Equity-informed brain care bundles similarly can be developed to assess proactive and reactive neuroprotective intervention outcomes. Gene–environment interactions will influence the dynamic neural exposome across each person’s lifespan. More effective therapeutic options can shift intergenerational neurodevelopmental trajectories to improve neurologic and mental health for entire communities. Combining biological, social, and structural determinants determine the direction of vulnerability or resilience pathways based on time-sensitive shared healthcare decisions. Two clinical vignettes ground this theoretical framework with fetal-neonatal neurology practice experiences. Emphasis on fragmented care, limited genomic screening, structural inequity, and uncorrected environmental exposures diminish preventable neurological and maternal outcomes across generations. We propose five implementation recommendations: dismantle structural barriers to integrate care; redesign training around transdisciplinary competency frameworks; realign payment structures to incentivize coordinated care; reorient research priorities with integrated care models; and develop measurable metrics of integrated maternal-child brain health. Artificial intelligence-assisted monitoring and learning health system platforms offer infrastructural elements to enable equitable intervention scaling across diverse clinical settings. Implementation of this framework across each lifespan will reduce intergenerational burdens of neurological and mental health disorders to sustain global brain health equity.

## Introduction: women’s health integrated with global brain health for children through matresensce

Optimal life-course healthcare decisions begin with knowledge of a woman’s and her partner’s childhood and reproductive experiences before and during each planned pregnancy. These choices significantly influence health across the lifespan for themselves and their children. This narrative review presents perspectives specifically focused on achieving more equitable life-course brain health principles and practice. Effective implementation requires shared knowledge among all stakeholders who will drive practical changes across the lifespan to improve global outcomes. These changes require healthcare bundles which have been defined as a set of three to five evidence-based interventions designed to assess the quality and outcome of medical care (www/IHS.org). This methodology will be applicable to brain care bundles as proactive and reactive intervention choices are considered. Information acquired for this review have used indexed peer-reviewed selections, prioritizing sources published within the last 10 years. This discussion is supplemented by relevant seminal older papers and policy documents. The information shared will contribute to a greater understanding of equitable healthcare efforts. Improvements in global brain health must prioritize the decolonization of public health delivery with increased benefits to those in the Global South by establishing partnerships with the Global North. These actions can be augmented by effective use of artificial intelligence (1). Critical thinking with agency (2, 3) will help all participants promote clinical decisions based on comprehensive knowledge transfer to build consensus. This learning process will more successfully combine forethought, implementation, self-management and learning through mindfulness and adaptation (4). More effective structured decisions can be offered during each clinical encounter across developmental and aging scenarios. Measurable framework components will generate necessary data inputs which feed into larger learning health systems designed to optimize brain health at the population level. Future research designs will optimize practice guidelines to initially improve women’s brain health for themselves and their children starting during each pregnancy (5, 6). Life-course brain health interventions will more inclusively support women, children and families (7). Shared decisions for women and their partners optimally require transdisciplinary care that begins before each pregnancy. These contributions will more effectively contribute to brain health delivery over each and successive generations. Advancing health advocacy for women who do not experience pregnancy must always remain a high priority to more successfully lower the burden of life-course brain disorders for all participants in social networks (8).

Neurologic and mental health educational reforms require systematic healthcare priorities that apply cultural neuroscience perspectives to examine global interplay involving culture, brain function and behavior (9). Developmental cultural neuroscience (10) examines the intersection of developmental psychology, cross-cultural psychology, and neuroscience across brain maturation and aging. Children’s and adolescents’ psychological and behavioral adjustments will more successfully shape brain structure and function into senescence. Critical-sensitive periods of neuroplasticity across the lifespan are further influenced by social concerns within globally relevant cultural contexts. Methodological approaches that merge cultural neuroscience principles with developmental processes offer opportunities to overcome challenges by using cross-cultural observations to measure psychological and biological processes (11). Assessments of fetal and childhood brain development that use genetic and neuroimaging biomarkers exemplify how cultural effects on women’s reproductive and pregnancy health initially influence subsequent life-course brain health (12). Overcoming time pressures, financial incentives, academic competitiveness and other cultural barriers help strengthen transdisciplinary critical thinking to improve clinical practices by all stakeholders.

Neuroprotective interventions for women before conception influence their children’s lifecycle health from prenatal through childhood into their adulthood. Six recommended pillars of brain health have been primarily emphasized for adult interventions (13). This approach would be more effective when instituted for girls and women during their reproductive years from menarche through menopause which has been defined as matresence (14). Positive or negative encounters will impact their children’s developing brain during the first 1,000 days from conception during which the first critical-sensitive period of neuroplasticity influences brain health with lifecycle effects (15). Maternal physical activity, nutrition and sleep quality for example contribute to fetal brain development (16). Suboptimal sleep during pregnancy detrimentally alters hippocampal volumes and connectivity which will be critical for life-long learning and memory (17). Maternal stress and suboptimal behaviors, particularly as part of mental health disorders negatively impact maternal-placental-fetal triad health resulting in adverse fetal neurodevelopment (18–20). Inflammatory effects impair maternal systemic interactions following maternal hypothalamic–pituitary adrenal axis dysfunction that result in adverse fetal stressor responses. Fetal demise, uteroplacental dysfunction, neonatal encephalopathy and childhood neurologic and mental health disorders more likely can ensue following pregnancies during which diseases and adversities have been experienced (21, 22). Abnormal neurodevelopmental trajectories result in anomalous or destructive brain lesions, sometimes detected by current fetal or neonatal neuroimaging technologies (19). Negative maternal postpartum depression and anxiety consequences will require time-sensitive interventions to preserve brain health of women and their children (23). Subsequent pregnancy challenges unfortunately can trigger negative behavioral responses based on previous complications which has been referred to as biological embedding (24).

The pre-conception time-period therefore offers a unique opportunity to introduce proactive neuroprotective interventions to sustain brain health for women during their pregnancy that will benefit their unborn children. Beneficial treatment responses more likely can be achieved as prenatal communicable and noncommunicable diseases are confronted. Infectious/inflammatory, hypertensive, metabolic, autoimmune disorders and mental health disorders each or collectively impair interconnected exposomes by altering gene–environment interactions that reduce maternal-placental-fetal triad health (25). Functional brain connectomes of their children will have completed crucial stages of segregation and integration involving neuronal connectivity during the first critical 1,000 days (26). Preventive neurology strategies can help mitigate disease occurrence and severity expressed as a range of childhood and adulthood neurologic disorders. Earlier efforts strengthen life cycle rescue and reparative interventions into senescence (27).

Providers continually are challenged by limited diagnostic capabilities as prospective neuroprotective options are considered. Outcomes will depend on individual resilience or vulnerability to disease and adversity. Current practices unfortunately treat maternal health and child neurodevelopment as separate domains. Fragmented care consequently is offered which diminishes important diagnostic results. Maternal and pediatric providers (e.g., obstetricians, neonatologists, neurologists, pediatricians, mental health providers, nurses, midwives) presently maintain siloed focus within their respective fields of expertise which reflect their respective certification requirements. Systematic reviews of cost-utility analyses reported only one-third of perinatal interventions included health outcomes that considered pregnant women together with their children (28, 29). The critical prevention window during the first 1,000 days does not currently receive necessary integrated health care applied across the life span. This represents a failure to capture the full societal value of integrated brain health interventions, particularly during the first critical neuroplasticity period following conception until the child’s age at 2 years.

Care bundles as proposed over a quarter century ago by the United States Institute of Health Care Improvement consist of at least three interventions when evaluating outcomes. Neuroscience knowledge by all stakeholders can identify transdisciplinary care paths to more effectively select proactive brain care bundles that begin before conception (30). These actions help improve shared healthcare decisions by prospective parents with physicians, nurses, midwives, and doulas as more aggressive neuroprotective choices are required throughout a problematic pregnancy. Greater health knowledge will empower women to contribute to their brain health to benefit their unborn children. Monitoring ferritin, glucose, nutrition, blood pressure and mental health using personal devices exemplify artificial intelligence methodologies with machine learning strategies to improve preventive maternal health care. These interventions are particularly important for women in communities where limited resources exist or who live geographically distant from medical facilities. Data sharing with their providers improves neuroprotective choices, calibrated to each woman’s health history before and during each pregnancy. Greater awareness regarding the full spectrum of available health innovations can be chosen by women, their partners and healthcare providers during first critical steps to achieve more effective solutions. Information dissemination through communications can be updated using responsible social media content, booklets, or electronic digests (for example, womenshealthdigest.org) that describe how public health strategies can reach a greater proportion of intended audiences.

Clinical fetal-neonatal neurology scenarios in the next two sections highlight the need to develop effective brain care bundles for women and children to sustain or restore brain health. These case histories were strategically selected to provide real-world clinical context that stresses a life-course approach derived from accurate storytelling by women and their family members. The developmental origins of health and disease concept is emphasized as future life-course preventive, rescue and reparative intervention options are designed that will benefit a greater percentage of targeted populations.

## Prenatal and neonatal brain care bundles are needed

This first maternal-child dyad is representative of low-risk pregnancies in both high and low-middle income countries. Subsequent unexpected fetal distress is often first detected during parturition by fetal heart pattern abnormalities. This woman received level one maternal health care from her healthcare providers who assumed maternal wellness starting at conception based on her preconception medical history. Normal prenatal surveillance testing described maternal and fetal well-being as pregnancy advanced across three trimesters (31). A neonatal male was vaginally delivered without the need for instrumentation. He required limited resuscitative interventions given transient depression recorded as a lower one-minute Apgar score of 3 with rapid improvement to 7 by 5 min of life. He was later assigned a modified Sarnat stage 2 score based on clinical expressions of irritability and hypertonia noted in the delivery suite (32). This assessment was supported by an arterial cord blood gas that documented a pH of 7.15, pCO_2_ of 48, a bicarbonate level of 15 with a base deficit of −10. Three days of therapeutic hypothermia were offered based on his examination findings in the context of a mixed respiratory and metabolic acid–base profile. His early neonatal hypertonia persisted over the next 24 h of life. No documentation of multi-systemic dysfunction, drug withdrawal, intracranial hemorrhage, meningitis, or pain syndromes excluded acute reasons associated with neonatal hypertonia (33). Antepartum maternal-placental-fetal triad disease pathways were alternatively considered. Chronic fetal brain subcortical motor pathway dysfunction over at least 3–5 days duration following cortical injuries have been described in fetal primate and rabbit pre-clinical models (34–38). Placental-cord examinations were not requested given the low-risk prenatal surveillance status followed by the child’s initial rapid recovery with neonatal resuscitation. Routine neonatal metabolic screening was negative for specific disorders. He exhibited an age-appropriate neonatal neurological examination at 2 weeks of life when assessed for discharge. Given his earlier neonatal encephalopathic presentation, pediatric neurology surveillance was offered. Poor state regulation with excessive irritability subsequently was observed over his first month of life which persisted throughout infancy. These behaviors were reported to the child’s pediatric providers based primarily on mother’s observations given father’s frequent absence given his employment responsibilities. She reported depressed mood and anxiety to her primary care providers who prescribed psychotropic medications and behavioral mental health interventions to treat postpartum depression. Facial dysmorphic features were later identified during the child’s infancy. Multi-domain developmental delays on psychometric testing documented language delay and poor socialization skills with observed stereotypies. Autistic spectrum disorder was provisionally considered by 2 years which was later confirmed at 4 years of age. Normal microarray and Fragile X studies were reported. At 5 years of age, he presented with acute clinical signs of lymphocytic leukemia. More specific genomic testing documented a chromosome 11 deletion associated with Jacobsen syndrome. He died within the year from medical complications of cancer. Mother required ongoing treatment for chronic depression and anxiety. Parents also sought family counseling while attempting to resolve persistent marriage difficulties. They did not attempt further pregnancies or pursue adoption.

Standard genetic screening for this woman’s low-risk pregnancy consisted of first trimester genetic screening for aneuploidy, guided by the parents’ healthy preconception history without known inherited familial diseases. More extensive reproductive or early pregnancy genetic testing may have identified the child’s specific chromosome 11 deletion during fetal life. Universal preconception or early pregnancy genetic testing protocols using whole exome and high through-put genomic sequencing however are not currently recommended for routine maternal care (39). Neonatal metabolic-genetic screening provides obligatory postnatal testing for comparatively rare inherited disorders. Falsely negative prenatal and neonatal genetic surveillance unfortunately do not identify children who later express childhood neurologic sequalae attributable to prenatal inherited disorders. Bioethical decisions continue to be debated whether more comprehensive testing should be performed (40, 41). Treatment protocols using genetically designed medications (42) have been proposed prior to the appearance of clinical signs of Jacobsen syndrome based on a genetically-engineered rodent disease model (43). Blockage of genetic regulatory abnormalities has been demonstrated for this chromosomal deletion designed for this animal model. Future proactive treatments before this child’s phenotypic expressions of disease could hypothetically avoid or lessen his sequalae expressed as autism and cancer. Attention to complex gene–environment interactions (44) during early developmental time periods with preemptive testing would encompass the full spectrum of developmental disorders such as children exhibiting cerebral palsy. Integrated use of preventive, rescue reparative intervention choices need to address inherited and acquired etiologies (45). Family decisions regarding subsequent pregnancy planning together with prenatal and neonatal treatments consequently might offer more effective treatments during earlier stages of a brain disorder. Rigorous bio-ethically driven protocols will be required to achieve information-informed shared decisions. Future healthcare policies that improve children’s outcomes will also contribute to lower life-course risks for depression more likely expressed by women and sustain more positive marital relationships beyond the women’s years of matrescence (46).

## Brain care bundles are needed through childhood into adulthood

The second dyad discusses more complex intragenerational and transgenerational effects that contribute to brain and mental health disorders for the child and parents. Intersectionality (47) is highlighted which influences health choices by women and partners that affect their children. A young adult black woman and her parents lived in a resource-limited American urban community. This woman’s mother previously experienced a high-risk pregnancy after she conceived her daughter while experiencing maternal obesity with insulin resistance which required obstetrical interventions consisting of weight reduction and serial serum glucose control management using Metformin. Despite these prenatal interventions, her daughter was born preterm at 34-weeks’ gestational age without fetal distress or encephalopathy. Placental-cord examinations were not performed. This child exhibited intellectual and socialization deficits during early childhood. Polycystic ovarian syndrome subsequently was diagnosed during adolescence comprised of insulin-dependent diabetes, obesity, hypothyroidism and dysmenorrhea. Her father had been an army veteran stationed state-side who later was self-employed as a house painter. Paints and solvents were stored and prepared in the family home. These commercial products have been reported to heighten risks for polycystic ovarian syndrome (48). Environmental toxin exposures during military service also have been reported for veterans (49), compounded by increased risks for toxin exposures of their families who live in socioeconomic-challenged communities (50).

Given their daughter’s complicated endocrinological history combined with her cognitive challenges to maintain health care compliance, consistent birth control could not be maintained. She experienced an unplanned pregnancy while living with her parents. Obstetrical and maternal-fetal medicine specialty providers managed her high-risk pregnancy with close parental supervision. Their daughter nonetheless experienced worsening multi-systemic disease complications, principally consisting of poor diabetic control and excessive weight gain superimposed on pre-pregnancy obesity (i.e., BMI > 40). Her poor health literacy and compliance despite her parent’s vigilance during prenatal care contributed to the unexpected home delivery of a 24-week gestational age male presenting after massive vaginal bleeding. Apgar scores were unavailable when the transport team arrived to treat this preterm neonate’s encephalopathic state, presumably following a massive abruptio placenta. Multi-systemic complications of prematurity required prolonged neonatal intensive care for this child, including management of intraventricular hemorrhage with bilateral intraparenchymal extension and progressive ventriculomegaly. Reduced placental size with maternal and fetal malperfusion lesions with acute hemorrhagic lesions were described on perinatal histopathology which have been correlated with pregnancies of women with polycystic ovarian syndrome (51). The mother died shortly after her hospital discharge from cerebral edema resulting in herniation secondary to acute uncontrolled diabetic ketoacidosis. The maternal grandparents assumed their grandson’s guardianship. He later experienced significant multi-domain delays including intractable epilepsy. An individualized educational program was maintained throughout his school years into early adulthood, supplemented by multiple therapy interventions beyond school hours. The maternal grandmother later experienced cognitive decline associated with a series of cerebrovascular events beginning during her seventh decade which contributed to her death. The paternal grandfather developed Parkinson’s disease and was unable to care for his grandson, requiring residential care until his death. Their adult grandson presently lives at a chronic healthcare facility with support from public funding. Adverse outcomes for the parents and child will be discussed, emphasizing life-course social determinants of health (52).

Social ecological theory illustrates the breakdown of healthcare for this second case study which adversely affected multiple system levels (52, 53) including brain health (54). At microsystem level, adverse childhood experiences, family toxin exposures and health literacy limitations compromised health behaviors practiced by the maternal grandmother and her daughter. At the mesosystem level, fragmented healthcare delivery presented real-world challenges to coordinate obstetrical, endocrinological, and social services for this Black family who lived in a resource-deprived urban neighborhood with greater toxin exposures. At the exosystem level, community poverty and healthcare deserts further limited access to timely and effective specialized care. At the macrosystem level, structural inequities based on race and social-economic class created negative healthcare conditions that persist across successive generations despite those families who live in high-income countries. Black women and children in particular experience higher rates of prematurity, fetal demise and maternal mortality (55). Systems theory suggests individual interventions remain insufficient given toxic stressor interplay that operates simultaneously across multiple levels for families as exemplified by this clinical scenario. Coordinated responses cannot be optimally offered given current healthcare conditions in communities where adequate healthcare resources are potentially available, as sought by these biological parents who were motivated to seek optimal prenatal care for their daughter and her unborn son. Life-course theory underscores how this grandmother’s previous compromised health experiences during her younger years of matrescence. This established conditions for temporal amplification of system failures across generations. This began with the birth of the parents’ preterm daughter who later experienced significant cognitive and mental health disorders prior to conception in the context of a complex endocrinological disease. Adverse life-course effects to their grandson’s brain health occurred given his condition of extreme prematurity at 24-weeks’ gestation. Grandmother experienced her own cognitive decline resulting from multiple stroke events that are more likely experienced by women than men given the chronic adverse effects from metabolic syndrome (56, 57). Men such as the paternal grandfather more likely experience neurodegenerative diseases such as Parkinson’s Disease (58, 59), possibly contributed by toxin exposures during his military service followed by employment as a house painter. Life course risks for brain and mental health diseases resulted after adverse exposome effects to parents and children. Application of the developmental origins of health and disease concept (60, 61) requires recognition of intersectoral factors that include sex-specific, racial and socioeconomic vulnerabilities across the lifespan.

## Gene–environment interactions define a dynamic neural exposome

Effective prenatal and neonatal brain care bundles require applications of the developmental origins of health or disease perspective as complex gene–environment interactions are later expressed. Development of time-sensitive biomarkers can facilitate more effective interventions during optimal developmental junctures. Non-linear and context-dependent dynamic trajectories of brain development during the first 1,000 days require small well-timed and targeted interventions during critical neuroplasticity time periods. Effects will be later amplified resulting in large-scale neurodevelopmental shifts throughout childhood. Early life interventions therefore will better sustain adult brain health into senescence. Neuroprotective strategies that address prenatal and neonatal disease pathways require knowledge of acute, subacute and chronic stages of neurodegeneration that potentially impair structure and function with time-dependent expressions by the dynamic neural exposome (62).

Knowledge of integrative neuroscience by all stakeholders can contribute to more accurate diagnostic choices, beginning with relatively low-cost reproductive and pregnancy health care decisions. Attention to early dietary treatments of fetal and neonatal microbiomes for example (63, 64) contribute to favorable connectome trajectories that favor brain health across the lifespan with continued wellness interventions (15). Prenatal maternal dysbiosis alternatively has been implicated with childhood neurodevelopmental and adult-onset neurodegenerative disorders. Early microbiome disruption negatively primes the immature immune system to express metabolic vulnerabilities that increase risks for adult brain and mental health diseases (65–68).

Fetal anomalous and destructive brain lesions identified with genetic disorders (69) remain important to offer accurate diagnostic and prognostic information for parental counseling and family planning. Given the complexity of interrelated genetic and acquired disease factors, however, many neurologic and mental health disorders are below detection thresholds for timely diagnoses using current structural, physiologic, or genetic tests. These limitations underestimate optimal selection of maternal health care interventions despite identification of increased risks in vulnerable populations. Long intervals often separate disease onset from phenotypic expression before or after birth (70). Interdisciplinary developmental neuroscience helps narrow the knowledge-practice gap to potentially introduce more effective brain care bundles during reproductive and pregnancy time-periods (15).

**Figure 1:**
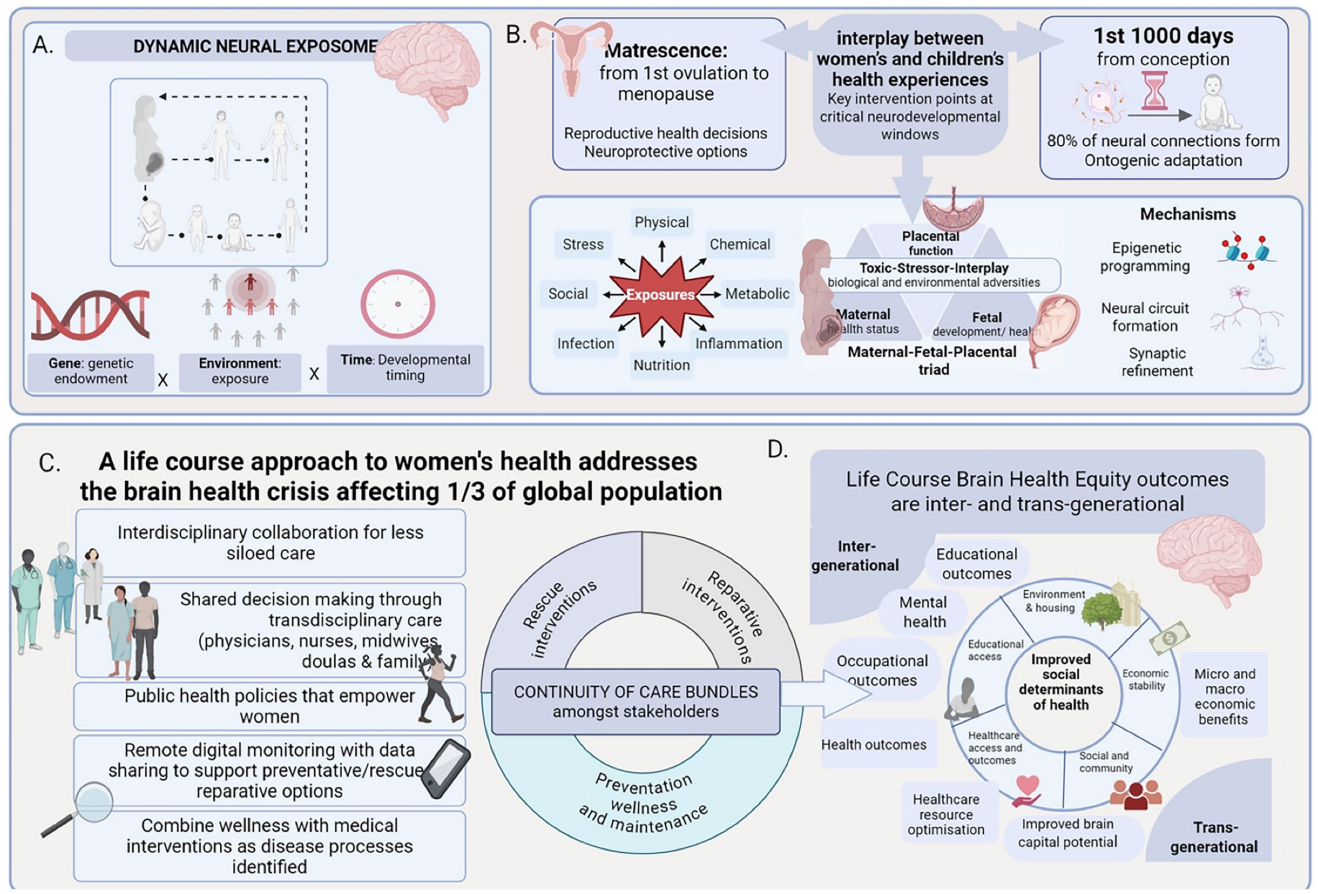
This framework demonstrates how critical thinking applied to women’s health care decisions during critical neurodevelopmental windows can achieve brain health equity across generations. **(A)** The dynamic neural exposome emerges from gene–environment-time interactions during neurocritical periods in both the mother’s and her child life during matrescence, with critical impact during the first 1,000 days when 80% of brain circuitry forms. **(B)** Multiple exposures affect the maternal-placental-fetal triad through toxic stressor interplay, mediated by epigenetic programming, neural circuit formation, and synaptic refinement. **(C)** A life-course approach requires interdisciplinary collaboration delivering continuity of care bundles (preventive and wellness maintenance, rescue, and reparative interventions) supported by empowering public health policies. **(D)** Outcomes span inter-generational (educational, mental and physical health, occupational) and trans-generational (economic stability, improved social determinants, reduced healthcare resource utilization) benefits, addressing the global brain health crisis affecting one-third of the world’s population.

The complexity of endogenous and exogenous factors associated with the human exposome has been termed toxic stressor interplay with cumulative influences across the lifespan (15, 71) (Figure 1A). Brain disorders such as postpartum depression as described for the woman in the first case study represent multiple maternal mental health illnesses that can impair the child’s fetal brain without prenatal clinical detection (61). Following maternal hypothalamic–pituitary–adrenal axis dysfunction, release of excessive corticosteroid and noradrenergic substances contribute to increased risks to acquire anomalous and/or destructive fetal brain lesions. Postnatal negative gene–environment interactions continue to exacerbate neurologic and mental health disorders by combined vulnerability from familial inheritability and multisystemic diseases further amplified by adverse childhood experiences such as poverty and poor family dynamics (72–74). The field of environmental neuroscience (75), exemplified by xenobiotic exposures and climate change demonstrate how complex exogenous and endogenous risks further diminish maternal-placental fetal (MPF) triad health followed into childhood and perpetuated throughout adulthood (Figure 1B).

Studies that correlate placental pathological lesions with neurodevelopmental outcomes provide mechanistic understanding of an integrated assessment of multiple factors associated with perinatal pathology that can be applied to fetal-neonatal neurology diagnoses (15). Maternal-placental fetal triad health effects correlate with three representative and inter-related placental disease pathways, maternal immune activation (60, 76, 77), ischemic placental syndrome (78–80), and maternal-fetal inflammatory responses (81, 82). Fetal brain disorders are derived from individual or collective expressions of these three disease pathways. An international perinatal pathology consensus report described four broad placental lesion categories associated with reduced neurodevelopmental outcomes, identified as maternal-fetal inflammatory responses, maternal or fetal malperfusion, chronic villitis and villous dysmaturity (83). Re-analyses of these criteria will re-enforce the importance of perinatal pathology as novel prenatal interventions are proposed (84, 85). Greater knowledge of placenta-cord pathology during formal fetal-neonatal neurology training has been stressed (15). However, this subject continues to remain undervalued, in part given lack of access to experienced perinatal pathological examinations. Placental dysfunction or dysregulation create bidirectional risks that alter fetal brain development with negative maternal caregiving behaviors that result in impaired maternal priming. This will be compounded by neurodevelopmental vulnerabilities as early disruptive postnatal maternal–infant interactions occur. A more accurate diagnostic approach will target a greater number of high-risk neonates who later express brain disorders. These actions will better enable providers to offer proactive neuroprotective interventions prior to phenotypic expressions to improve outcomes before as well as after delivery (86).

The dynamic neural exposome expresses time-dependent gene–environment interactions initially over the first 1,000 days with foundational brain effects across the lifespan (15, 71, 87). Parental genetic endowment combined with trimester-specific and postnatal post-translational or epigenetic child-related diseases or adversities require time-sensitive neuroprotective strategies. These efforts will more effectively address the global public health crisis of neurological and mental health disorders expressed over the lifespan (87, 88) (Figure 1C). Gene–environment interactions start before each pregnancy (Figure 1D). Transgenerational plasticity (89, 90) extend effects from exposome stressors that affect each nuclear family during each generation with future societal influences. Gene–environment interactions start before each pregnancy. Reducing adverse stress responses of offspring will result by more effectively controlling human environments that avoid or reduce maladaptive neuronal mechanisms across the lifespan.

The maternal-placental-fetal triad should be more comprehensively viewed as a series of complex dynamic networks that simultaneously operate across different in utero timescales. Nested ultradian, circadian, and infradian rhythmic effects have relatively more influences across successive gestational stages. Maternal chronobiologic rhythms directly influence fetal development and long-term offspring health effects. Lack of synchrony among rhythms may disrupt each other, affecting the maternal-placental-fetal triad which impacts intervention effectiveness (91, 92). Monitoring maternal and fetal rhythms with sleep medicine evaluations help identify women who otherwise experience unrecognized higher risk pregnancies. Simple interventions such as encouraging regular sleep, meal timing, and light–dark exposures during pregnancy help restore disrupted synchrony similar to adult brain health wellness practices (13). Prospective decisions by all stakeholders can subsequently benefit from iterative reassessments, as additional knowledge is acquired with adjusted life-course intervention choices.

Systematic barriers need to be dismantled that have diminished integrative healthcare efforts. Reconfiguring postnatal healthcare can achieve more positive responses by support of the maternal-child dyad and family throughout childhood beginning with prenatal brain health interventions for the maternal-placental-fetal triad. Support for evidence-based maternal care includes provider knowledge, training, and service motivations. Effective multi-level coordination, leadership and effective communication maintain trust by women and their partners (93). Critical thinking decisions must apply “fast-thinking-slow thinking” (94) strategies to fetal-neonatal neurology principles and practice. These actions connect knowledge siloes among multiple disciplines to select the most effective life-course interventions (15). Shared information among obstetrics/gynecology, maternal-fetal medicine, nursing, midwifery, neonatology, behavioral-developmental pediatrics, genetics, and mental health specialists will improve informed integrated clinical decisions with parents regarding brain health for the woman and her child. Diagnostic acumen is further augmented by information interpreted by life-course trained neuroradiology, neurophysiology, perinatal pathology and genetics specialists. Data processing and interpretation also benefit from engineering, computational science, biophysics, epidemiologic-statistical and bioethical-humanistic expertise. Interdisciplinary collaborations among all these types of providers should include formal fetal-neonatal training. This knowledge can be applied to career-long clinical practice that also strengthen educational and research activities to benefit everyone they serve.

Fetal and neonatal test interpretations remain constrained by sensitivity and specificity limitations. Most fetal neurology consultants primarily provide diagnoses based primarily on genetic and neuroimaging information. Many prenatal brain diseases however remain unrecognized given conventional diagnostic testing. By applying developmental neuroscience knowledge during consultations, fetal neurology specialists can offer more accurate diagnostic and prognostic guidance. Consideration of the biological process termed ontogenetic adaptation helps distinguish favorable from unfavorable developmental responses as more advanced stages of brain development are expressed following earlier responses (95). Time-sensitive brain health care remains challenging given that only a symptomatic fetal or neonatal minority will exhibit neurological disorders requiring neonatal neurocritical care. An unrecognized majority of children will express clinical signs over the first 2 years of age at which time rescue or reparative interventional strategies will more likely be chosen with reduced efficacy. Applying the science of uncertainty (96) can help stakeholders combine initial creative decisions that heuristically may be error-prone. More accurate re-analyses can be offered as additional information is acquired over the first 5,000 days before the child enters school (97).

## Childhood educational experiences prepare for adult brain health

Interventions continue as young people enter school. Important opportunities exist that can prevent, maintain or restore brain health when medical and social contextual information are mutually considered. Early identification of vulnerable mother-infant dyads through validated observational tools can help guide target interventions during critical preschool attachment periods to benefit formal education. Psychometric evaluations using different observational coding systems during variable assessment intervals of the mother and child’s psychological health have been described (98), correlated with the child’s neuroimaging findings (99). Childhood communicable and noncommunicable diseases and adversities threaten brain health, particularly during time-sensitive pediatric neurocritical care scenarios. Reassessments will require different diagnostic, therapeutic and prognostic choices in response to new or worsening neurologic and mental health disorders. The developmental origins of health and disease concept will help guide brain health care interventions as children process their school year experiences to maximize educational opportunities after graduation (15). This approach prioritizes comprehensive prenatal care (100) for the maternal-placental-fetal triad that helps preserve brain health throughout childhood into adulthood. This must be inclusive of the child’s formal education for life-course investment. Successful adult employment and a more meaningful quality of life can be attained and sustained into senescence (54, 101).

Identification of pre-school clinical signs or epidemiologic risk assessments helps formulate more effective mainstreamed educational or specialized scholastic interventions. These efforts are supported by the application of educational neuroscience (98, 99) principles that optimize every child’s academic and social performance. Educational plans require parent’s participation with information from neurologic, mental health and therapy-based providers who identified motor, language, cognitive and behavioral disorders or challenges for their child. Bidirectionality between neurologic and mental health as exemplified by childhood anxiety and depression are often associated with developmental disorders and epilepsy which further reduce scholastic performances. Educational brain bundles are therefore useful for teachers, strengthened by parental-family involvement to be maintained outside the school day. These interventions are particularly essential for all children particularly with those experiencing less functional neurodivergence who may not express permanent neurologic disorders. Educational and social interventions require longitudinal coordination across developmental transitions. These actions will ensure that early brain health investments translate into measurable improvements throughout higher education, vocational training and career selection. Success in the adult workplace will certainly more likely yield greater economic prosperity but more importantly result in an improved quality of life (54, 101). Such an approach requires systematic integration by connecting existing assessments across specialties. Rather than treating maternal health and child brain development as isolated domains, more effective interventions throughout adulthood can be offered.

Addressing structural, social and environmental drivers of health (Figure 1D) offer opportunities to reduce toxic stressor interplay that improve brain health for children and adolescents during their formal education. This is particularly relevant in communities where maternal and pediatric healthcare deserts present more formidable barriers to brain health equity (102). As highlighted by the discussion of the second clinical vignette, social ecological theory explains how individual health outcomes result from interactions across multiple system levels - microsystem (family), mesosystem (healthcare), exosystem (community resources including education), and macrosystem (policy environment). Greater success can be achieved with integrative strategies for women’s and children’s brain health practices that are implemented before adulthood. There will consequently be a greater likelihood of intragenerational and transgenerational benefits for women, children and families.

## Innovative artificial intelligence with machine learning technologies

Innovative technologies using artificial intelligence (AI) algorithms can assist stakeholders with more accurate decision making. Examples of AI diagnostic accuracy figures are cited below, recognizing the limitations of training based diversity, generalizability to low-resource settings, and regulatory status. Novel AI-powered solutions, nonetheless, can improve brain risk assessments starting during prenatal life. More timely interventions can prevent or lessen fetal brain injuries and reduce unnecessary medical interventions after birth. For example, AI models applied to fetal brain MRI presently have demonstrated accuracies ranging from approximately 85 to 99% depending on the task (103). The highest performances were observed using pathological classification for brain localization with slightly lower but robust results regarding brain tissue segmentation and placental disease detection. These models support the use of a complete image generation pipeline that includes preprocessing (i.e., artifact and motion correction), brain extraction, segmentation with gestational age prediction within approximately 1 week using advanced classification tasks.

Deep-learning algorithms can also be used for segmentation training to classify normal and abnormal fetal brain ultrasound images using standard axial planes based on heat maps for lesion localization (104). These algorithms help distinguish normal from abnormal fetal brain ultrasound images with reported 96.3% overall accuracy, achieve 96.9% sensitivity and 95.9% specificity to identify abnormal images, with precise localization of lesions in 61.6% of abnormal cases in this specific study.

Novel software can also perform AI-based assessment of fetal anatomy using abdominal ultrasound biometry, delineating appropriate planes for measuring head circumference, biparietal diameter, transcerebellar diameter, and other critical neurological parameters (105). This software currently demonstrates good to excellent reproducibility in fetal skull biometric measurements with reported assessment times that average 63 seconds which is amenable during busy office-based maternal imaging centers.

Some tools are presently in preliminary exploratory stages, requiring initial FDA clearance such as in the United States. For example, an AI-powered tool automatically can detect standard views on routine anatomy scans during second and third-trimester fetal heart ultrasound evaluations (106). This software will support antepartum fetal heart assessments when all recommended views are utilized, enabling exam completeness and consistency regardless of operator experience. These technological solutions more expeditiously can address risks for perinatal injury as the expected delivery date approaches to be applied during the intrapartum period. This new deep learning model shows promise by reporting rapid detection of fetal compromise derived from fetal heart rate signals that use invariant input length. This system has achieved approximately 25% reduction in the time required to detect fetal distress when compared to current state-of-the-art methods. These tools will enable obstetrical providers to consider alternative intrapartum interventions for higher risk fetuses than have been previously identified.

Postnatal examples have also been described using AI include analyses of infant movements using smartphone videos (107), submitted by parents for their providers’ interpretation. These technologies can facilitate a more definitive diagnosis of motor abnormalities using automated general infant movement assessments which correlate with increased cerebral palsy risks (108). These technologies can accurately track movements and detect abnormal or absent movements based on parents’ recordings in 76% of cases, compared with subsequent assessments by experienced clinicians. Another diagnostic AI application incorporates hardware into a pocket-sized wireless system in the neonatal intensive care unit to detect neonatal seizures (109). This system potentially facilitates automated seizure detection using multiple electrode recordings, even in resource-challenged medical centers. These advanced AI algorithms will support analyses by comparing a child’s real-time neurophysiologic data with published peer-reviewed research results to choose antiepileptic medications for treatment of neonatal seizures.

Integration of innovative technologies such as the chosen examples described include a wide variety of stand-alone artificial intelligence algorithms or AI-powered solutions incorporated into smartphones and specialized hardware devices. These represent novel opportunities to design and implement more effective prenatal and neonatal brain care bundles (30). With clinical validation and adoption into clinical practice, such systems have the potential to enhance preventive and rescue interventions with earlier detection strategies to treat fetal and early life brain pathologies. Their applications can enable proactive risk management to advance scalability of care. Such technologies collectively provide a framework to transition from reactive diagnoses to comprehensive proactive maternal-fetal health surveillance that supports improved care coordination. This consequently will empower women to make better informed decisions during pregnancy to improve their children’s brain health with adult benefits.

## Knowledge of neuroplasticity with neurodegeneration is influenced by intersectionality

Attention to life-course neuroplasticity provides important perspectives when choosing intervention strategies throughout development and aging. Recognition of cellular and molecular changes that promote recovery from disease expressions must consider different stages of neurodegeneration (62) in relation to genetic and acquired vulnerabilities (110, 111). Long prodromal time periods often separate embryonic and fetal brain disease onset from postnatal phenotypic expressions with current diagnostic limitations. Primary, secondary and tertiary neurodegenerative changes will contribute to functional neuroadaptation or dysfunctional maladaptation across advancing life-cycle time scales that begin during pregnancy. Time-sensitive interventions can lessen effects from apoptosis, necroptosis, autophagy, protein homeostasis, inflammation, microgliosis and astrogliosis (112). Gene–environment interactions responsible for eight hallmarks of cellular neurodegeneration have been associated with neurodegenerative disease pathways. These include pathological protein aggregation, synaptic and neuronal network dysfunction, aberrant proteostasis, cytoskeletal abnormalities, altered energy homeostasis with mitochondrial dysfunction, DNA and RNA defects, inflammation, and neuronal cell death (113). These findings are predominately based on preclinical studies using mature and aging animal brain models (59, 113, 114). More recent attention has now been applied to the fetus and neonate (115–117). Models of neurodegeneration will require the perspective of intersectionality, integrating sex, gender, ethnic, racial and socioeconomic factors for global applications (118–120). Women and their partners consequently will more likely benefit using proactive neurotherapeutic approaches that influence the first 1,000 days of childhood brain health with substantial life-course brain health benefits.

## Shifting the prognostic curve to more favorable outcomes for brain health

Emphasis on women’s health promotes brain health equity for themselves and their children, resulting in more reassuring normal outcomes or functional neurodiversity into adulthood after positive neuroadaptation (121). This approach will enable a greater number of children to achieve stronger educational skills which can be applied to more successful adult workplace performances with a better quality of life (54). Longitudinal non-randomized cohort studies such as an Icelandic research collaborative (122) demonstrated improved adult brain wellness after preventive neurology interventions. This investigative approach is being globally advocated by multiple professional neurological organizations (123–127). This Nordic study will require validation with more heterogenous global populations in other nations that introduce intersectoral factors into study populations based on sex, gender, race, ethnicity, resources and geographic differences. Global life-course brain and mental health remain goals for all communities (128) to address the public health crisis involving over one-third of the world’s population with brain disorders (129). Equitable health begins with women who contribute to their improved brain health across the lifecycle (130) with multi-generational benefits to their children and families.

Low-cost proactive early life interventions sustained over time can potentially reduce the prevalence of permanent neurologic disorders rather than reliance on a single therapeutic choice (131). Breastfeeding with longer durations for example have demonstrated dose-dependent relationships when initiated immediately after birth and maintained through early childhood. Improved IQ scores and scholastic achievements have been reported, contributing to reduced risks for behavioral problems at older school ages (132, 133). While debates continue involving confounding effects from observational studies, beneficial effects are possibly mediated through mechanisms that include optimal fatty acid utilization, immunomodulation, and beneficial microbial establishment. Maternal oral probiotic supplementation offer similar possible associations with decreased levels of breastmilk inflammatory biomarkers, reduced infant fecal microbiome variations, and altered infancy recognition memory responses as reported in a pilot observational study (134). Breast feeding when combined with skin-to skin contact currently constitute recommended developmental interventions that can be initiated immediately after birth (15, 30). These measures are applicable particularly in resource-limited communities to offer more positive short and long-term impacts on structural and functional brain health (135–138). With these early life neuroprotective measures, later-life preventive, rescue and reparative choices (Figure 1C) can potentially avoid or mitigate debilitating effects of adult neurologic disorders. Reduced incidence of adult cerebrovascular, neurodegenerative, and mental health disorders using neurotherapeutic care pathways before and during the first 1,000-days will also be particularly helpful for women as they transition from matrescence into senescence. Older women more frequently experience specific adult neurologic and mental health disorders than men such as dementia, stroke and depression (130).

## Implementation framework

The previous fetal-neonatal neurology case studies illustrate different forms of system failure which prompt a crucial question regarding innovative framework implementation. Can we imagine future clinical scenarios in which key interventions can more effectively improve outcomes by focusing on women maternal-child dyads? The following explanations are therefore compelling and challenge current health policy priorities with a focus on improved life-course brain health strategies. Case 1 demonstrates how this proposed framework for brain health should not only focus on prevention but strive to coordinate optimal earlier diagnoses with timely interventions. Life-course consequences of an inherited disease can be averted based on risk assessments during ontogeny prior to appearance of clinical signs. Case 2 reflects more societal-driven structural inequities not easily addressed by only interdisciplinary collaborations. Major alterations in health care systems are required that offer more accurate systematic identification of higher risks with intergenerational ramifications for population brain health. Health care requires bio-ethical responsibilities by addressing intersectionality to reduce structural, social, and environmental inequities. Transdisciplinary healthcare interventions encompass continuity of brain care bundles by all stakeholders who strive to maintain equitable and pragmatic brain health neuroprotective choices. Person-specific interventions across successive generations require support by universally applied public health measures (Figure 1D).

Effective brain health care delivery requires specific and actionable interventions that are applicable across stakeholder groups. The most formidable challenge to overcome entails efforts to improve universal health and well-being through foundational transformations involving multiple systems rather than improving existing ones (139). We propose three immediate implementation strategies that address healthcare fragmentation beginning during matrescence. A fourth longer-term strategy will require fundamental medical education reform. Just as biological systems exhibit sensitive periods for developmental modification, healthcare systems have critical windows during which more effective transformation can be achieved. Implementation should target each opportunity to offer improvements regarding healthcare-related education of all stakeholders. Changes in formal residency training and accreditation requirements for providers, institutional accreditation for healthcare delivery systems and regularly scheduled health policy reviews need to be addressed from improvements to be achieved.

**Figure 2:**
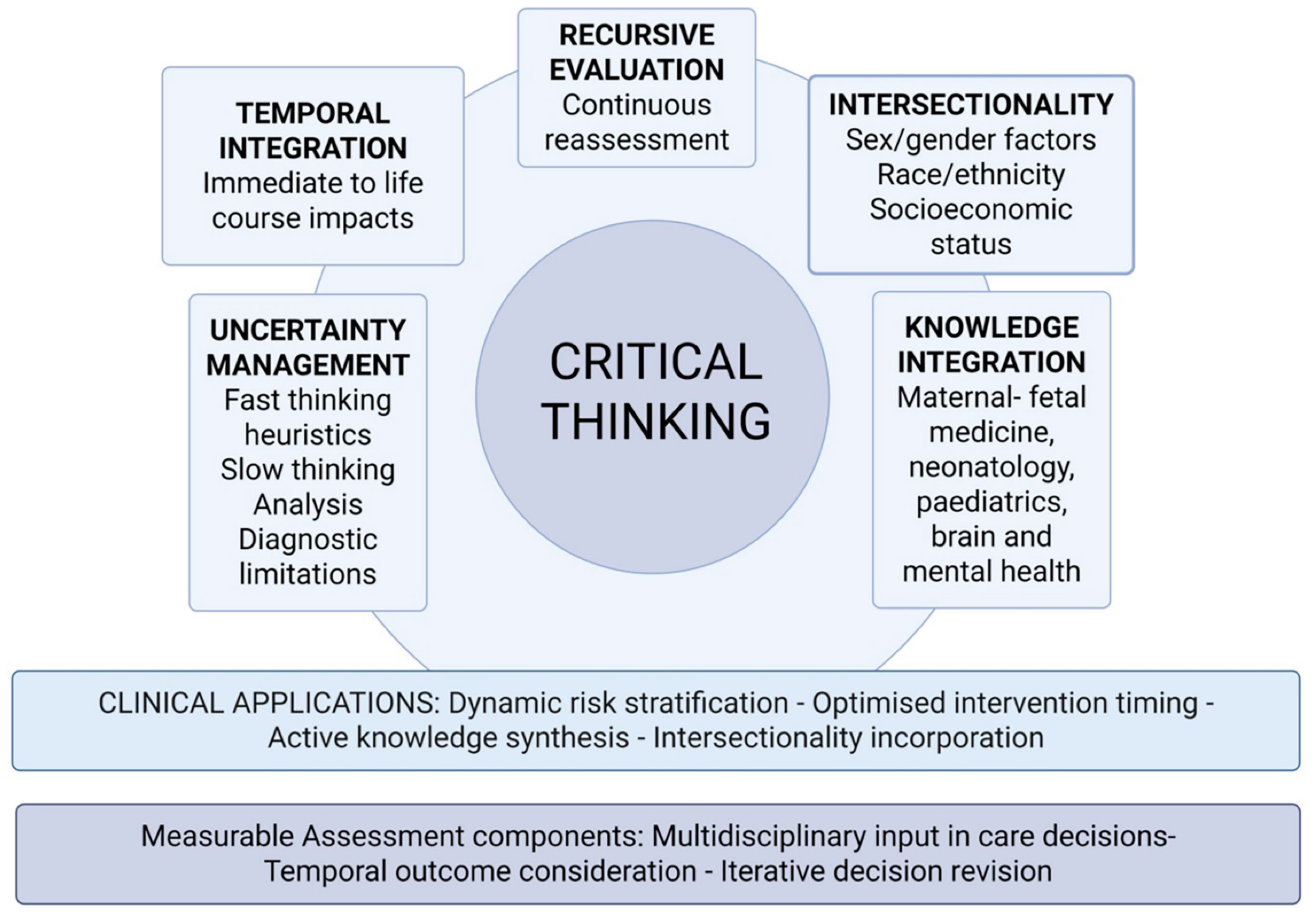
This depicts an operational framework for critical thinking. An individual-level decision-making framework integrates five core components (temporal integration, knowledge synthesis, uncertainty management, intersectionality assessment, and recursive evaluation) to guide clinical encounters. The framework includes specific clinical applications and measurable assessment components that enable healthcare systems to implement, train, and evaluate critical thinking approaches.

**Figure 3:**
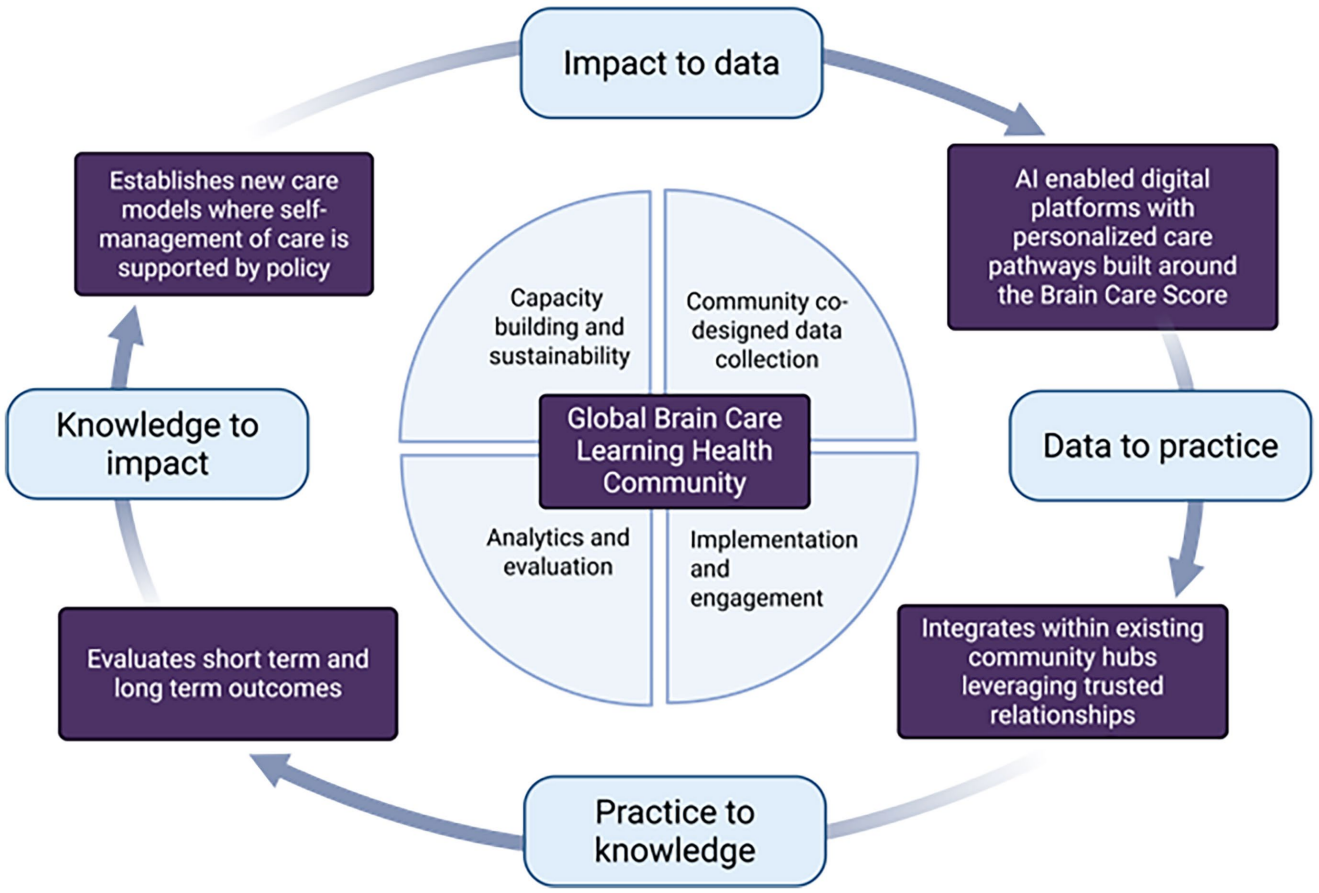
The global brain care coalition learning health system model (45). These individual decisions aggregate to inform population-level learning health systems. This figure depicts a global brain care learning health community that applies data to practice that is based on knowledge that impacts person-specific and public health brain care efforts. Both short and long-term outcomes are evaluated using serial analytics, assisted by artificial intelligence enabled digital platforms. These strategies apply intersectionality to offer brain care equity across the life cycle. This systems-level framework aggregates individual critical thinking decisions (see Figure 2) to create population-level learning and evidence generation for brain health optimization across communities.

Critical thinking decisions by each stakeholder as documented through the framework in Figure 2 aggregate to inform the Global Brain Care Coalition Learning Health System model (Figure 3). This effort will create a continuous feedback loop between clinical practice and population-level learning. These complementary frameworks operate synergistically: an effective structured decision-making process can be offered for individual encounters while the learning health systems provide the infrastructure for scalable successful interventions for populations to generate new evidence that better informs future clinical decisions. These systematic changes help enable approaches that anticipate revisions. Provisional solutions are offered that can be re-analyzed and prioritized as new information becomes available. Clinical protocols link maternal health assessments with fetal brain health risk stratification starting with routine reproductive and prenatal transdisciplinary care encounters among stakeholders. Training programs are prepared for providers to recognize interdisciplinary synergy among specialty domains to be extended after birth into childhood ad adulthood. Payment structures offer reimbursements for proactive coordinated care based on documented long-term outcomes into old age rather than offering fragmented services. Technological integration enables the monitoring and timely intervention of this framework by leveraging the advantages of artificial intelligence with machine learning to share, process and apply large and complicated data sets that improve health care delivery.

Learning health systems (LHS) represent a transformative approach for integrated data-driven decision-making. Continuous improvements can be applied to pediatric care across all biological systems that is inclusive of the continuity of childhood to adulthood brain health care. Prior work in pediatric learning health systems (140) have demonstrated that LHS can accelerate the translation of research findings into practice to improve outcomes for children. The Global Brain Care Coalition (GBCC) exemplifies a brain care-centric LHS, operating as a dynamic learning community dedicated to the discovery, design, development, and deployment of scalable interventions (globalbraincare.org). Central to the GBCC model is the Brain Care Score (141), a multidimensional metric that guides personalized prevention and treatment strategies. As shown in Figure 3, this model fosters iterative feedback across stakeholders, uniting providers, families, and policy leaders to optimize brain health throughout the life course.

The stratified approach described above ensures that implementation can proceed with established interventions while simultaneously generating evidence for novel approaches. These steps will transform the current healthcare system into a LHS that continuously optimizes brain health interventions based on real-world outcomes. Establishing integrated research capabilities will mirror clinical integration to create seamless data linkage between maternal health records and longitudinal child neurodevelopmental outcomes derived from improved health outcomes for women. Learning health system platforms that incorporates women’s and children’s health would capture real-time data for integrated health care applications to improve outcomes. Standardized protocols will ensure consistent measurement across settings. This infrastructure would support pragmatic trials that randomize health systems rather than individual patients, enabling rigorous evaluation of integrated care models. Every clinical encounter becomes an opportunity for evidence generation. This process would create a virtuous cycle to improve care, while simultaneously building evidence for further optimization to reduce or avoid a vicious cycle.

A longer-term transformational strategy will guarantee continued brain health benefits for successive generations. The blueprint for modern medical education established by the 1910 Flexner Report advanced scientific rigor but embedded structural limitations that persist today (142). By consolidating training within a small number of scientifically elite institutions, this report disproportionately eliminated schools serving black students and women. Clinical reasoning was anchored in a white male biomedical default with organized curricula around specialty disciplines rather than integrated clinical problems consisting of intersectoral factors. The legacy of siloed specialty training, marginalization of intersectionality, and the subordination of relational and social reasoning to reductionist science remain significantly unreformed in current accreditation and postgraduate training frameworks into the 21st century.

For fetal-neonatal neurology training with career-long learning, a training model organized within separate disciplines will not adequately educate clinicians to develop integrative reasoning required to preserve or restore maternal-child brain health. Obstetricians, neonatologists, neurologists, mental health, nursing and therapy-based providers each develop comprehensive competence certification examinations that remain confined within their domains. Structured exposure to interdisciplinary critical thinking that applies to first 1,000 days is required (15). Reform does not simplistically require an expansion of the existing curricula. Substantial reorganization must prioritize transdisciplinary clinical problems (143, 144), intersectionality-informed frameworks (145), and competency-based assessment to reward integrative thinking (146). Positive developments over the latter 20th century have included evidence-based results from longitudinal birth cohort studies (124, 147), global interdisciplinary professional organizations (87, 130, 148), and university-community academic hubs (149) that suggest how this reorganization can be achievable when driven by educational forethought and planning. Non-randomized longitudinal intervention research protocols are needed based on causal inference methodologies (150, 151) that support institutional commitments focused on improvements in public health (152).

## Recognition of limitations for future directions

This review has several limitations that should be considered when interpreting its conclusions as plans are proposed. As a narrative review, the literature selection reflects the authors’ collective expertise and thematic judgment which introduces the possibility of selection bias. The wide breadth of topics addressed include reproductive and pregnancy gene–environment interactions, placental pathology, microbiome science, chronobiology, AI-assisted monitoring, and educational neuroscience. This complex approach inevitably means that the depth of critical appraisal and strength of supporting evidence across domains will vary. The evidence cited is predominantly derived from primarily high-income country settings. The feasibility and generalizability of the proposed clinical and training framework in low- and middle-income country contexts, as well as in communities within high-income countries where healthcare deserts persist will require specific investigations. Finally, the five implementation recommendations are framework-level proposals. Prospective real-world data regarding their clinical effectiveness, educational impact, and cost-effectiveness do not yet exist.

## Concluding recommendations: healthcare policies support life-course brain health for each individual

The global burden of neurological and mental health disorders affects over one-third of the world’s population, exposing critical gaps regarding current healthcare system approaches to brain health care delivery. Maternal health and child neurodevelopment efforts are treated as separate domains, missing opportunities for coordinated interventions during the most influential developmental periods. The interplay between maternal and child health care must consider bidirectional relationships with cumulative advantages or disadvantages throughout a woman’s years of matrescence when one or multiple pregnancies may be experienced. Brain health across the lifespan beyond the years of reproduction will affect women as well as their adult children. Time-dependent gene–environment interactions continue to alter a dynamic neural exposome for persons across the lifespan (25, 71). Early interventions for the maternal-child dyad identify and treat those with disease or risk conditions (153).

This narrative review suggests that every pregnancy be treated as a brain health opportunity. Teaching critical thinking applied to transdisciplinary care helps foster connections among knowledge siloes across medical specialties. Shared decisions with the persons they serve will optimize brain health outcomes. Perspectives have been proposed that recognize the woman, her partner, and the maternal-placental-fetal triad as an integrated bio-social system early in each lifecycle. Continuity of brain care bundles initially offer preventive, rescue, and reparative interventions during the 1,000 days. Biological and environmental toxic stressor interplay requires comprehensive and timely reassessments and interventions across childhood into adulthood. Selected case studies were presented that highlight current healthcare fragmentation which contributes to missed diagnostic and therapeutic opportunities with suboptimal outcomes. A more integrated approach will improve early detection with more effective neuroprotection for neurologic and mental health disorders that otherwise remain unrecognized until later in life.

Implementation of these perspectives require the following five recommendations: (1) Systematic dismantling of barriers are essential to promote integrative care. (2) Training programs require organizational structures that prepare providers to recognize knowledge connections across specialty domains. (3) Payment structures must incentivize coordinated care over fragmented services. (4) Research priorities must focus on evaluation of integrated care models rather than investigations of isolated interventions. (5) Develop metrics of success through systematic maternal-child brain health integration based on measurable improvements in neurodevelopmental outcomes, cognitive performance, and economic returns while reducing lifetime healthcare burdens with an improved quality of life. Inequities cannot be addressed through healthcare coordination alone but also require parallel policy interventions. Public health policies must strive to offer person-specific applications, anticipating effects on brain health across each and successive lifespans. More successful brain health capital strategies in the workplace (54) can be prioritized based on maternal and child healthcare choices during reproductive, pregnancy and childhood time periods in preparation for brain health throughout adulthood (15, 30). Success will require collaborative commitments from individual clinicians, healthcare institutions, and policy makers who work together with persons in their communities. These healthcare improvements will transform fragmented care delivery into coordinated intergenerational brain health optimization.

## References

[1] Kakraba S Agyemang EF Srivastav SK. Cognitive sovereignty and decolonial public health: reclaiming epistemic authority in the global AI era. Front Public Health. (2026) 14:2026. doi: 10.3389/fpubh.2026.1785170 PMC13062185 41971282

[2] Prosperi M Min JS Bian J Modave F. Big data hurdles in precision medicine and precision public health. BMC Med Inform Decis Mak. (2018) 18:139. doi: 10.1186/s12911-018-0719-2, 30594159 PMC6311005

[3] Helou MA DiazGranados D Ryan MS Cyrus JW. Uncertainty in decision making in medicine: A scoping review and thematic analysis of conceptual models. Acad Med: Lippincott Williams Wilkins. (2020) 95:157–65. doi: 10.1097/ACM.0000000000002902, 31348062 PMC6925325

[4] Romero M Isaac G Barma S Girard M-A Heiser L Critical Thinking, Creativity, and Agency for the Development of Regenerative Cultures. Conference paper 1RMBAM Nice France (2023)

[5] Temmerman M Khosla R Laski L Mathews Z Say L. Women’s health priorities and interventions. BMJ [Br Med J]. (2015) 351:h4147. doi: 10.1136/bmj.h4147, 26371215

[6] Siristatidis C Karageorgiou V Vogiatzi P. Current issues on research conducted to improve women's health. Healthcare (Basel). (2021) 9:92–103. doi: 10.3390/healthcare9010092, 33477390 PMC7830703

[7] Halfon N Russ SA Schor EL. The emergence of life course intervention research: optimizing health development and child well-being. Pediatrics. (2022) 149:e2021053509C. doi: 10.1542/peds.2021-053509C PMC9847410 35503314

[8] Lassi ZS Wade JM Ameyaw EK. Stages and future of women's health: A call for a life-course approach. Womens Health (Lond). (2025) 21:17455057251331721. doi: 10.1177/17455057251331721, 40258196 PMC12035259

[9] Kwon JY Wormley AS Varnum MEW. Changing cultures, changing brains: A framework for integrating cultural neuroscience and cultural change research. Biol Psychol. (2021) 162:108087. doi: 10.1016/j.biopsycho.2021.108087, 33781802

[10] Qu Y Telzer EH. Developmental cultural neuroscience: Progress and Prospect. Handbook Culture Biol. (2017):465–87.

[11] Chiao JY. Developmental aspects in cultural neuroscience. Dev Rev. (2018) 50:77–89. doi: 10.1016/j.dr.2018.06.005, 30778272 PMC6377197

[12] Lowe P Lee E Macvarish J. Growing better brains? Pregnancy and neuroscience discourses in English social and welfare policies. Health Risk Soc. (2015) 17:15–29. doi: 10.1080/13698575.2014.994479, 25810690 PMC4353307

[13] Jaqua E Biddy E Moore C Browne G. The impact of the six pillars of lifestyle medicine on brain health. Cureus. (2023) 15:e34605. doi: 10.7759/cureus.34605, 36883088 PMC9985951

[14] Orchard ER Rutherford HJV Holmes AJ Jamadar SD. Matrescence: lifetime impact of motherhood on cognition and the brain. Trends Cogn Sci: Elsevier Ltd. (2023) 27:302–16. doi: 10.1016/j.tics.2023.06.002 PMC9957969 36609018

[15] Scher MS Eyre H Donn S Roberts JM Msall ME Salafia CM . An interdisciplinary fetal neonatal neurology collaborative promotes integrative life-course brain health. Front Neurol. (2025) 16:1725289. doi: 10.3389/fneur.2025.1725289, 41573402 PMC12819184

[16] Na X Raja R Phelan NE Tadros MR Moore A Wu Z . Mother's physical activity during pregnancy and newborn's brain cortical development. Front Hum Neurosci. (2022) 16:943341. doi: 10.3389/fnhum.2022.943341, 36147297 PMC9486075

[17] Nevarez-Brewster M Demers CH Mejia A Haase MH Bagonis MM Kim SH . Longitudinal and prospective assessment of prenatal maternal sleep quality and associations with newborn hippocampal and amygdala volume. Dev Cogn Neurosci. (2022) 58:101174. doi: 10.1016/j.dcn.2022.101174, 36375383 PMC9661438

[18] Li J Olsen J Vestergaard M Obel C. Attention-deficit/hyperactivity disorder in the offspring following prenatal maternal bereavement: a nationwide follow-up study in Denmark. Eur Child Adolesc Psychiatry. (2010) 19:747–53. doi: 10.1007/s00787-010-0113-9, 20495989

[19] Wu Y De Asis-Cruz J Limperopoulos C. Brain structural and functional outcomes in the offspring of women experiencing psychological distress during pregnancy. Mol Psychiatry. (2024) 29:2223–40. doi: 10.1038/s41380-024-02449-0, 38418579 PMC11408260

[20] Mandl S Alexopoulos J Doering S Wildner B Seidl R Bartha-Doering L. The effect of prenatal maternal distress on offspring brain development: A systematic review. Early Hum Dev. (2024) 192:106009. doi: 10.1016/j.earlhumdev.2024.106009, 38642513

[21] Shen Q Zhong W Wang X Fu Q Mao C. Associations between pregnancy loss and common mental disorders in women: a large prospective cohort study. Front Psychol. (2024) 15:2024. doi: 10.3389/fpsyt.2024.1326894 PMC10957736 38525260

[22] Herbert D Young K Pietrusińska M MacBeth A. The mental health impact of perinatal loss: A systematic review and meta-analysis. J Affect Disord. (2022) 297:118–29. doi: 10.1016/j.jad.2021.10.026, 34678403

[23] eClinicalMedicine. Safeguarding maternal mental health in the perinatal period. EClinicalMedicine. (2024) 71:102663. doi: 10.1016/jeclinm.2024.102663 38813440 PMC11133788

[24] Thomas JC Letourneau N Bryce CI Campbell TS Giesbrecht GF. Biological embedding of perinatal social relationships in infant stress reactivity. Dev Psychobiol. (2017) 59:425–35. doi: 10.1002/dev.21505, 28220490

[25] Scher MS. Interdisciplinary fetal-neonatal neurology training applies neural exposome perspectives to neurology principles and practice. Front Neurol. (2023) 14:1321674. doi: 10.3389/fneuro.2023.1321674 38288328 PMC10824035

[26] Li Q Xia M Zeng D Xu Y Sun L Liang X . Development of segregation and integration of functional connectomes during the first 1,000 days. Cell Rep. (2024) 43:114168. doi: 10.1016/j.celrep.2024.114168, 38700981

[27] Sun L Zhao T Liang X Xia M Li Q. Human lifespan changes in the brain's functional connectome. Nat Neurosci. (2025) 28:891–901. doi: 10.1038/s41593-025-01907-4, 40181189

[28] Lamsal R Yeh EA Pullenayegum E Ungar WJ. A systematic review of methods and practice for integrating maternal, fetal, and child health outcomes, and family spillover effects into cost-utility analyses. PharmacoEconomics. (2024) 42:843–63. doi: 10.1007/s40273-024-01397-5, 38819718 PMC11249496

[29] Abel L Dakin H Cai T McManus RJ McNiven A Rivero-Arias O. How are maternal and fetal outcomes incorporated when measuring benefits of interventions in pregnancy? Findings from a systematic review of cost-utility analyses. Health Qual Life Outcomes. (2024) 22:75. doi: 10.1186/s12955-024-02293-4, 39256866 PMC11389402

[30] Scher MS Eyre HA Adalat S Hartikainen K Berk M Ibanez A . Childhood health through matrescence empowers women to sustain life-course brain health. NPJ Womens Health. (2026) 4:16. doi: 10.1038/s44294-026-00135-w, 33069006

[31] Menard MK Kilpatrick S Saade G Hollier LM Joseph GF Jr Barfield W . Levels of maternal care. Am J Obstet Gynecol. (2015) 212:259–71. doi: 10.1016/j.ajog.2014.12.030, 25620372

[32] Sarnat HB Flores-Sarnat L Fajardo C Leijser LM Wusthoff C Mohammad K. Grading scale for neonatal encephalopathy (Sarnat & Sarnat 1976): 45 year update proposal. Pediatr Neurol. (2020) 113:75–9. doi: 10.1016/j.pediatrneurol.2020.08.014, 33069006

[33] Scher MS. Neonatal hypertonia: II. Differential diagnosis and proposed neuroprotection. Pediatr Neurol. (2008) 33:373–80. doi: 10.1016/pediatrneurol.2008.09.009 19027581

[34] Lawrence DG Kuypers HGJM. The functional organization of the motor system in the monkey. I. The effects of bilateral pyramidal lesions. Brain J Neurol. (1968) 91:1–14. 10.1093/brain/91.1.1 4966862

[35] Lawrence DG Kuypers HGJM. The functional organization of the motor system in the monkey. II. The effects of lesions of the descending brain-stem pathways. Brain J Neurol. (1968) 91:15–36. 10.1093/brain/91.1.15 4966860

[36] Drobyshevsky A Derrick M Luo K Near-Term Fetal Hypoxia-Ischemia in Rabbits MRI Can Predict Muscle Tone Abnormalities and Deep Brain Injury. Stroke (2012) 43:2757–63. 10.1161/STROKEAHA.112.653857 PMC3458142 22829546

[37] Drobyshevsky A. Concurrent decrease of brain white matter tracts' thicknesses and fractional anisotropy after antenatal hypoxia-ischemia detected with tract-based spatial statistics analysis. J Magnetic Resonance Imag: JMRI. (2017) 45:829–38. doi: 10.1002/jmri.25407, 27505718 PMC6109450

[38] Synowiec S Lu J Yu L Goussakov I Lieber R Drobyshevsky A. Spinal hyper-excitability and altered muscle structure contribute to muscle hypertonia in newborns after antenatal hypoxia-ischemia in a rabbit cerebral palsy model. Front Neurol. (2019) 9:1183. doi: 10.3389/fneur.2018.01183, 30705663 PMC6344443

[39] National Academies of Sciences E, Medicine. Newborn Screening in the United States: A Vision for Sustaining and Advancing Excellence. Washington, DC: The National Academies Press (2025). 40966374

[40] Deprest J Toelen J Debyser Z. The fetal patient -- ethical aspects of fetal therapy. Facts Views Vis Obgyn. (2011) 3:221–7. 24753868 PMC3991449

[41] Spencer SJ Fullerton SM. Population genomic screening: ethical considerations to guide age at implementation. Front Genet. (2022) 13:2022. doi: 10.3389/fgene.2022.899648 PMC9577139 36267415

[42] Akshoomoff N Mattson S Grossfeld P. Evidence for autism spectrum disorder in Jacobsen syndrome: identification of a candidate gene in distal 11q. Genet Med Official J American College of Medical Genetics. (2014) 17:143–148. doi: 10.1038/gim.2014.86 25058499

[43] Nakamura T Arima-Yoshida F Sakaue F Nasu-Nishimura Y Takeda Y Matsuura K . PX-RICS-deficient mice mimic autism spectrum disorder in Jacobsen syndrome through impaired GABAA receptor trafficking. Nat Commun. (2016) 7:10861. doi: 10.1038/ncomms10861, 26979507 PMC4799364

[44] Miao J Song G Wu Y Hu J Wu Y Basu S . PIGEON: a statistical framework for estimating gene–environment interaction for polygenic traits. Nat Hum Behav. (2025) 9:1654–68. doi: 10.1038/s41562-025-02202-9, 40410536 PMC12496094

[45] Basu AP Low K Ratnaike T Rowitch D. Genetic investigations in cerebral palsy. Dev Med Child Neurol. (2025) 67:177–85. doi: 10.1111/dmcn.16080, 39208295 PMC11695794

[46] Sinkewicz M Rostant O Zivin K McCammon R Clarke P. A life course view on depression: social determinants of depressive symptom trajectories over 25 years of Americans’ changing lives. SSM Popul Health. (2022) 18:101125. doi: 10.1016/j.ssmph.2022.101125, 35664926 PMC9160836

[47] Subramaniapillai S Galea LAM Einstein G de Lange A-MG. Sex and gender in health research: intersectionality matters. Front Neuroendocrinol. (2024) 72:101104. doi: 10.1016/j.yfrne.2023.101104, 39492521

[48] Sharma P Bilkhiwal N Chaturvedi P Kumar S Khetarpal P. Potential environmental toxicant exposure, metabolizing gene variants and risk of PCOS-A systematic review. Reprod Toxicol. (2021) 103:124–32. doi: 10.1016/j.reprotox.2021.06.005, 34126208

[49] Goldman SM Weaver FM Stroupe KT Cao L Gonzalez B Colletta K . Risk of Parkinson disease among service members at marine Corps Base camp Lejeune. JAMA Neurol. (2023) 80:673–81. doi: 10.1001/jamaneurol.2023.1168, 37184848 PMC10186205

[50] Johnston J Cushing L. Chemical exposures, health, and environmental justice in communities living on the Fenceline of industry. Curr Environ Health Rep. (2020) 7:48–57. doi: 10.1007/s40572-020-00263-8, 31970715 PMC7035204

[51] Hochberg A Mills G Volodarsky-Perel A Nu TNT Machado-Gedeon A Cui Y . The impact of polycystic ovary syndrome on placental histopathology patterns in in-vitro fertilization singleton live births. Placenta. (2023) 139:12–8. doi: 10.1016/j.placenta.2023.05.015, 37290292

[52] Lopez M Ruiz MO Rovnaghi CR Tam GKY Hiscox J Gotlib IH . The social ecology of childhood and early life adversity. Pediatr Res. (2021) 89:353–67. doi: 10.1038/s41390-020-01264-x, 33462396 PMC7897233

[53] Stokols D. Translating social ecological theory into guidelines for community health promotion. Am J Health Promot. (1996) 10:282–98. 10159709 10.4278/0890-1171-10.4.282

[54] Ibanez A Melloni L Świeboda P Hynes W Ikiz B Ayadi R . Neuroecological links of the exposome and one health. Neuron. (2024) 112:1905–10. doi: 10.1016/j.neuron.2024.04.016, 38723637 PMC11189719

[55] Toval CA Darivemula SM Wilson TD Conklin JL Young OM. Interventions to mitigate pregnancy-related mortality and morbidity in Black birthing people: a systematic review. American J Obstetrics Gynecol MFM. (2024) 6:101464. doi: 10.1016/j.ajogmf.2024.101464, 39147362

[56] Garovic VD White WM Vaughan L Saiki M Parashuram S Garcia-Valencia O . Incidence and long-term outcomes of hypertensive disorders of pregnancy. J Am Coll Cardiol. (2020) 75:2323–34. doi: 10.1016/j.jacc.2020.03.028, 32381164 PMC7213062

[57] Miller EC Conley P Alirezaei M Wolfova K Gonzales MM Tan ZS . Associations between adverse pregnancy outcomes and cognitive impairment and dementia: a systematic review and meta-analysis. Lancet Healthy Longev. (2024) 5:100660. doi: 10.1016/j.lanhl.2024.100660, 39675366 PMC11726346

[58] Bianco A Antonacci Y Liguori M. Sex and gender differences in neurodegenerative diseases: challenges for therapeutic opportunities. Int J Mol Sci. (2023) 24:6354. doi: 10.3390/ijms24076354, 37047320 PMC10093984

[59] Sakowski SA Koubek EJ Chen KS Goutman SA Feldman EL. Role of the Exposome in neurodegenerative disease: recent insights and future directions. Ann Neurol. (2024) 95:635–52. doi: 10.1002/ana.26897, 38411261 PMC11023772

[60] Braun AE Carpentier PA Babineau BA Narayan AR Kielhold ML Moon HM . “Females are not just ‘protected’ males”: sex-specific vulnerabilities in placenta and brain after prenatal immune disruption. eNeuro. (2019) 6:ENEURO.0358–19. doi: 10.1523/eneuro.0358-19.2019, 31611335 PMC6838689

[61] Monk C Lugo-Candelas C Trumpff C. Prenatal developmental origins of future psychopathology: mechanisms and pathways. Annu Rev Clin Psychol. (2019) 15:317–44. doi: 10.1146/annurev-clinpsy-050718-095539, 30795695 PMC7027196

[62] Marzola P Melzer T Pavesi E Gil-Mohapel J Brocardo PS. Exploring the role of neuroplasticity in development, aging, and neurodegeneration. Brain Sci. (2023) 13:1610. doi: 10.3390/brainsci13121610, 38137058 PMC10741468

[63] Puglisi CH Kim M Aldhafeeri M Lewandowski M Vuong HE. Interactions of the maternal microbiome with diet, stress, and infection influence fetal development. FEBS J. (2025) 292:1437–53. doi: 10.1111/febs.70031, 39988792 PMC11927046

[64] Catassi G Aloi M Giorgio V Gasbarrini A Cammarota G Ianiro G. The role of diet and nutritional interventions for the infant gut microbiome. Nutrients. (2024) 16:400. doi: 10.3390/nu16030400, 38337684 PMC10857663

[65] Lu J Claud EC. Connection between gut microbiome and brain development in preterm infants. Dev Psychobiol. (2019) 61:739–51. doi: 10.1002/dev.21806, 30460694 PMC6728148

[66] Hassib L de Oliveira CL Rouvier GA Kanashiro A Guimarães FS Ferreira FR. Maternal microbiome disturbance induces deficits in the offspring's behaviors: a systematic review and meta-analysis. Gut Microbes. (2023) 15:2226282. doi: 10.1080/19490976.2023.2226282, 37400971 PMC10321199

[67] Frerichs NM de Meij TGJ Niemarkt HJ. Microbiome and its impact on fetal and neonatal brain development: current opinion in pediatrics. Curr Opin Clin Nutr Metab Care. (2024) 27:297–303. doi: 10.1097/MCO.0000000000001028, 38488112 PMC10990016

[68] Eisen A Kiernan MC. The neonatal microbiome: implications for amyotrophic lateral sclerosis and other neurodegenerations. Brain Sci. (2025) 15:195. doi: 10.3390/brainsci15020195, 40002527 PMC11852589

[69] Lerman-Sagie T Hart AR. The fetal neurologist: strategies to improve training, practice, and clinical care. Dev Med Child Neurol. (2025) 67:1118–29. doi: 10.1111/dmcn.16301, 40101002 PMC12336404

[70] White F. Application of disease etiology and natural history to prevention in primary health care: a discourse. Med Princ Pract. (2020) 29:501–13. doi: 10.1159/000508718, 32422632 PMC7768156

[71] Kunikullaya UK. An integrated approach to understanding the effects of exposome on neuroplasticity. Behav Brain Res. (2025) 485:115516. doi: 10.1016/j.bbr.2025.115516 40024484

[72] Mamun A Biswas T Scott J Sly PD McIntyre HD Thorpe K . Adverse childhood experiences, the risk of pregnancy complications and adverse pregnancy outcomes: a systematic review and meta-analysis. BMJ Open. (2023) 13:e063826. doi: 10.1136/bmjopen-2022-063826, 37536966 PMC10401231

[73] Bhutta ZA Bhavnani S Betancourt TS Tomlinson M Patel V. Adverse childhood experiences and lifelong health. Nature Med: Nature Res. (2023) 29:1639–48. doi: 10.1038/s41591-023-02426-0 37464047

[74] Qiu A Shen M Buss C Chong YS Kwek K Saw SM . Effects of antenatal maternal depressive symptoms and socio-economic status on neonatal brain development are modulated by genetic risk. Cereb Cortex. (2017) 27:3080–92. doi: 10.1093/cercor/bhx065, 28334351 PMC6057508

[75] Berman MG Stier AJ Akcelik GN. Environmental neuroscience. Am Psychol. (2019) 74:1039–52. doi: 10.1037/amp0000583, 31829683

[76] Han VX Patel S Jones HF Dale RC. Maternal immune activation and neuroinflammation in human neurodevelopmental disorders. Nat Rev Neurol. (2021) 17:564–79. doi: 10.1038/s41582-021-00530-8, 34341569

[77] Hall MB Willis DE Rodriguez EL Schwarz JM. Maternal immune activation as an epidemiological risk factor for neurodevelopmental disorders: considerations of timing, severity, individual differences, and sex in human and rodent studies. Front Neurosci: Front Media SA. (2023) 17:1135559. doi: 10.3389/fnins.2023.1135559 PMC10133487 37123361

[78] Kwiatkowski S Kwiatkowska E Rzepka R Torbe A Dolegowska B. Ischemic placental syndrome--prediction and new disease monitoring. J Maternal-Fetal Neonatal Med: Official J European Association of Perinatal Medicine, the Federation of Asia and Oceania Perinatal Societies, Int Society of Perinatal Obstetricians. (2016) 29:2033–9. doi: 10.3109/14767058.2015.1072165, 26444581

[79] Harris LK Benagiano M D'Elios MM Brosens I Benagiano G. Placental bed research: II. Functional and immunological investigations of the placental bed. Am J Obstet Gynecol. (2019) 221:457–69. doi: 10.1016/j.ajog.2019.07.010, 31288009

[80] Brosens I Puttemans P Benagiano G. Placental bed research: I. The placental bed: from spiral arteries remodeling to the great obstetrical syndromes. Am J Obstet Gynecol. (2019) 221:437–56. doi: 10.1016/j.ajog.2019.05.044, 31163132

[81] Goldstein JA Gallagher K Beck C Kumar R Gernand AD. Maternal-fetal inflammation in the placenta and the developmental origins of health and disease. Front Immunol: Front Media SA. (2020) 531543. doi: 10.3389/fimmu.2020.531543 PMC7691234 33281808

[82] Kovács K Kovács ŐZ Bajzát D Imrei M Nagy R Németh D . The histologic fetal inflammatory response and neonatal outcomes: systematic review and meta-analysis. Am J Obstet Gynecol. (2024) 230:493–511.e3. doi: 10.1016/j.ajog.2023.11.1223, 37967697

[83] Khong TY Mooney EE Ariel I Balmus NCM Boyd TK Brundler MA . Sampling and definitions of placental lesions: Amsterdam placental workshop group consensus statement. Arch Pathol Lab Med. (2016) 140:698–713. doi: 10.5858/arpa.2015-0225-CC, 27223167

[84] Redline RW Roberts DJ Parast MM Ernst LM Morgan TK Greene MF . Placental pathology is necessary to understand common pregnancy complications and achieve an improved taxonomy of obstetrical disease. Am J Obstet Gynecol. (2023) 228:187–202. doi: 10.1016/j.ajog.2022.08.010, 35973475 PMC10337668

[85] Yaguchi C Ueda M Mizuno Y Fukuchi C Matsumoto M Furuta-Isomura N . Association of Placental Pathology with physical and neuronal development of infants: A narrative review and reclassification of the literature by the consensus statement of the Amsterdam placental workshop group. Nutrients. (2024) 16:1786. doi: 10.3390/nu16111786, 38892717 PMC11174896

[86] Upadhyay RP Taneja S Chowdhury R Dhabhai N. Child neurodevelopment after multidomain interventions from preconception through early childhood: the WINGS randomized clinical trial. JAMA. (2024) 331:28–37. doi: 10.1001/jama.2023.23727, 38165408 PMC10762577

[87] World Health Organization. Optimizing Brain Health Across the Life Course; a Position Paper. Geneva: World Health Organization (2022).

[88] Winter SF Walsh D Catsman-Berrevoets C Feigin V. National plans and awareness campaigns as priorities for achieving global brain health. Lancet Glob Health. (2024) 12:e697–706. doi: 10.1016/s2214-109x(23)00598-3, 38485433 PMC10951964

[89] Donelan SC Hellmann JK Bell AM Luttbeg B Orrock JL Sheriff MJ . Transgenerational plasticity in human-altered environments. Trends Ecol Evol. (2020) 35:115–24. doi: 10.1016/j.tree.2019.09.003, 31706627 PMC9440440

[90] Hellmann JK Sih A. Integrating social learning, social networks, and non-parental transgenerational plasticity. Trends Ecol Evol. (2025) 40:335–45. doi: 10.1016/j.tree.2024.12.001, 39755518

[91] Strohmaier S Devore EE Vetter C Eliassen AH Rosner B Okereke OI . Night shift work before and during pregnancy in relation to depression and anxiety in adolescent and young adult offspring. Eur J Epidemiol. (2019) 34:625–35. doi: 10.1007/s10654-019-00525-2, 31081539 PMC6548754

[92] Bates K Herzog ED. Maternal-fetal circadian communication during pregnancy. Front Endocrinol (Lausanne). (2020) 11:198. doi: 10.3389/fendo.2020.00198, 32351448 PMC7174624

[93] Dadich A Piper A Coates D. Implementation science in maternity care: a scoping review. Implement Sci. (2021) 16:16. doi: 10.1186/s13012-021-01083-6, 33541371 PMC7860184

[94] Tversky A Kahneman D. Judgment under uncertainty: heuristics and biases. Sci AAAS. (1974) 185:1124–31. 10.1126/science.185.4157.1124 17835457

[95] Bjorklund DF. "Ontogenetic adaptations". In: Encyclopedia of Evolutionary Psychological Science. In: Shakelford TK Weekes-Shakelford VA, Editors. Cham: Springer International Publishing (2016). p. 1–3.

[96] Moulder G Harris E Santhosh L. Teaching the science of uncertainty. Diagnosis (Berl). (2023) 10:13–8. doi: 10.1515/dx-2022-0045 36087299

[97] Draper CE Yousafzai AK McCoy DC. The next 1000 days: building on early investments for the health and development of young children. Lancet. (2024) 404:2094–116. doi: 10.1016/S0140-6736-(24)01389-8 39571589 PMC7617681

[98] Wilcox G Morett LM Hawes Z Dommett EJ. Why educational neuroscience needs educational and school psychology to effectively translate neuroscience to educational practice. Front Psychol. (2021) 11:618449. doi: 10.3389/fpsyg.2020.618449, 33519642 PMC7840578

[99] Matta C. Neuroscience and educational practice–a critical assessment from the perspective of philosophy of science. Educ Philos Theory. (2021) 53:197–211. doi: 10.1080/00131857.2020.1773801

[100] McDonald CR Weckman AM Wright JK Conroy AL Kain KC. Developmental origins of disease highlight the immediate need for expanded access to comprehensive prenatal care. Front Public Health. (2022) 10:2022. doi: 10.3389/fpubh.2022.1021901 PMC9730730 36504964

[101] Eyre HA Hynes W Ayadi R Manes F Swieboda P. Brain capital is crucial for global sustainable development. Lancet Neurol: Elsevier Ltd. (2024) 23:233–5. doi: 10.1016/S1474-4422(24)00031-0 38280391

[102] Brigance CLRJEDAOMMK Henderson Z. Nowhere to Go: Maternity Care Deserts Across the U.S. (Report No. 3). Arlington County, Virginia: March of Dimes (2022).

[103] Vahedifard F Adepoju JO Supanich M Ai HA Liu X Kocak M . Review of deep learning and artificial intelligence models in fetal brain magnetic resonance imaging. World J Clin Cases. (2023) 11:3725–35. doi: 10.12998/wjcc.v11.i16.3725, 37383127 PMC10294149

[104] Xie HN Wang N He M Zhang LH Cai HM Xian JB . Using deep-learning algorithms to classify fetal brain ultrasound images as normal or abnormal. Ultrasound Obstet Gynecol. (2020) 56:579–87. doi: 10.1002/uog.21967, 31909548

[105] Mlodawski J Zmelonek-Znamirowska A Mlodawska M Detka K Białek K Swiercz G. Repeatability and reproducibility of artificial intelligence-acquired fetal brain measurements (SonoCNS) in the second and third trimesters of pregnancy. Sci Rep. (2024) 14:25076. doi: 10.1038/s41598-024-77313-w, 39443660 PMC11500000

[106] BrightHeart. BrightHeart Obtains Third FDA Clearance and PCCP Approval, Becoming First to Offer One Integrated Solution for Real-Time Fetal Heart Documentation and CHD Detection. (2025). Available online at: www.businesswire.com

[107] Passmore E Kwong AL Greenstein S Olsen JE Eeles AL Cheong JLY . Automated identification of abnormal infant movements from smart phone videos. PLOS Digit Health. (2024) 3:e0000432. doi: 10.1371/journal.pdig.0000432, 38386627 PMC10883563

[108] Spittle AJ Marschik PB Adde L Badawi N. Towards universal early screening for cerebral palsy: a roadmap for automated general movements assessment. EClinicalMedicine. (2025) 86:103379. doi: 10.1016/j.eclinm.2025.103379, 40735348 PMC12304702

[109] Micheli C Thelen A De Vos M Dereymaeker A Haueisen J Fiedler P. Validation of an automated seizure detection procedure for Multi-Channel neonatal EEG. Appl Sci. (2026) 16:52. doi: 10.3390/app16010052, 30654563

[110] Fu H Hardy J Duff KE. Selective vulnerability in neurodegenerative diseases. Nat Neurosci. (2018) 21:1350–8. doi: 10.1038/s41593-018-0221-2, 30250262 PMC6360529

[111] Manuello J Min J McCarthy P Alfaro-Almagro F Lee S Smith S . The effects of genetic and modifiable risk factors on brain regions vulnerable to ageing and disease. Nat Commun. (2024) 15:2576. doi: 10.1038/s41467-024-46344-2, 38538590 PMC10973379

[112] Moujalled D Strasser A Liddell JR. Molecular Mechanisms of cell Death in Neurological Diseases.Cell Death and Differentiation (2021) 28: 2029–44. 10.1038/s41418-021-00814-y PMC8257776 34099897

[113] Wilson DM 3rd Cookson MR Van Den Bosch L Zetterberg H Holtzman DM Dewachter I. Hallmarks of neurodegenerative diseases. Cell. (2023) 186:693–714. doi: 10.1016/j.cell.2022.12.032, 36803602

[114] Wareham LK Liddelow SA Temple S Benowitz LI di Polo A Wellington C . Solving neurodegeneration: common mechanisms and strategies for new treatments. Mol Neurodegener. (2022) 17:23. doi: 10.1186/s13024-022-00524-0, 35313950 PMC8935795

[115] Pickler RH McGrath JM Reyna BA McCain N Lewis M Cone S . A model of neurodevelopmental risk and protection for preterm infants. J Perinat Neonatal Nurs. (2010) 24:356–65. doi: 10.1097/jpn.0b013e3181fb1e70, 21045616 PMC3494740

[116] Vancamp P Frapin M Parnet P Amarger V. Unraveling the molecular mechanisms of the neurodevelopmental consequences of fetal protein deficiency: insights from rodent models and public health implications. Biological Psychiatry Global Open Science. (2024) 4:100339. doi: 10.1016/j.bpsgos.2024.100339, 39040432 PMC11262180

[117] Martin LJ Lee JK Niedzwiecki MV Amrein Almira A Javdan C Chen MW . Hypothermia shifts neurodegeneration phenotype in neonatal human hypoxic–ischemic encephalopathy but not in related piglet models: possible relationship to toxic conformer and intrinsically disordered prion-like protein accumulation. Cells. (2025) 14:14. doi: 10.3390/cells14080586, 40277911 PMC12025496

[118] Kobayashi LC Peterson RL Yu X Avila-Rieger J Amofa-Ho PA Vila-Castelar C . Life course financial mobility and later-life memory function and decline by gender, and race and ethnicity: an intersectional analysis of the US KHANDLE and STAR cohort studies. Lancet Healthy Longev. (2024) 5:100613. doi: 10.1016/S2666-7568(24)00129-6, 39222645 PMC11472306

[119] Besser LM Fuentes AJ Zhang JN O’Shea DM Galvin JE. Intersectionality of gender with social determinants of health and asymptomatic Alzheimer's disease neuropathology. J Alzheimer's Dis. (2024) 102:110–8. doi: 10.1177/13872877241283823, 39497306 PMC11915083

[120] Misiura MB Butts B Hammerschlag B Munkombwe C Bird A Fyffe M . Intersectionality in Alzheimer's disease: the role of female sex and Black American race in the development and prevalence of Alzheimer's disease. Neurotherapeutics. (2023) 20:1019–36. doi: 10.1007/s13311-023-01408-x, 37490246 PMC10457280

[121] Steger JS Land BB Lemos JC Chavkin C Phillips PEM. Insidious transmission of a stress-related neuroadaptation. Front Behav Neurosci. (2020) 14:564054. doi: 10.3389/fnbeh.2020.564054, 33132859 PMC7571264

[122] Muller M Sigurdsson S Kjartansson O Gunnarsdottir I Thorsdottir I Harris TB . Late-life brain volume: a life-course approach. The AGES-Reykjavik study. Neurobiol Aging. (2016) 41:86–92. doi: 10.1016/j.neurobiolaging.2016.02.012, 27103521 PMC5751431

[123] Kolappa K Seeher K Dua T. Brain health as a global priority. J Neurol Sci. (2022) 439:120326. doi: 10.1016/j.jns.2022.120326 35777091

[124] Wagen AZ Coath W Keshavan A James SN Parker TD Lane CA . Life course, genetic, and neuropathological associations with brain age in the 1946 British birth cohort: a population-based study. Lancet Healthy Longev. (2022) 3:e607–16. doi: 10.1016/S2666-7568(22)00167-2, 36102775 PMC10499760

[125] Sabayan B Doyle S Rost NS Sorond FA Lakshminarayan K Launer LJ. The role of population-level preventive care for brain health in ageing. Lancet Healthy Longev. (2023) 4:e274–83. doi: 10.1016/s2666-7568(23)00051-x, 37201543 PMC10339354

[126] Eyre HA Stirland LE Jeste DV Reynolds CF III Berk M Ibanez A . Life-course brain health as a determinant of late-life mental health: American Association for Geriatric Psychiatry expert panel recommendations. Am J Geriatr Psychiatry. (2023) 31:1017–31. doi: 10.1016/j.jagp.2023.09.013, 37798224 PMC10655836

[127] Farina FR Bridgeman K Gregory S Crivelli L Foote IF Jutila OEI . Next generation brain health: transforming global research and public health to promote prevention of dementia and reduce its risk in young adult populations. Lancet Healthy Longev. (2024) 5:100665. doi: 10.1016/j.lanhl.2024.100665, 39718180 PMC11972554

[128] Hulshoff Pol HE Brouwer RM. Unique opportunities and challenges of longitudinal approaches in studying brain health and mental health. Neuron. (2025) 113:1858–61. doi: 10.1016/j.neuron.2025.04.029, 40409252

[129] Steinmetz JD Seeher KM Schiess N Nichols E. Global, regional, and national burden of disorders affecting the nervous system, 1990–2021: a systematic analysis for the global burden of disease study 2021. Lancet Neurol. (2024) 23:344–81. doi: 10.1016/S1474-4422(24)00038-3, 38493795 PMC10949203

[130] World economic forum. World Economic Forum in collabortion with the McKinsey Health Institute Closing the Women’s _ Health Gap 2024. (2024). p. 1–41.

[131] Sheth KN Dorsey ER Okun MS. Preventive neurology isn't a pill; it's a plan. JAMA Neurol. (2025) 83:97–98. doi: 10.1001/jamaneurol.2025.4445 41247706

[132] Bar S Milanaik R Adesman A. Long-term neurodevelopmental benefits of breastfeeding. Curr Opin Pediatr. (2016) 28:559–66. doi: 10.1097/mop.0000000000000389, 27386975

[133] Goldshtein I Sadaka Y Amit G Kasir N Bourgeron T Warrier V . Breastfeeding duration and child development. JAMA Netw Open. (2025) 8:e251540. doi: 10.1001/jamanetworkopen.2025.1540, 40126480 PMC11933992

[134] Gonia S Heisel T Miller N Haapala J Harnack L Georgieff MK . Maternal oral probiotic use is associated with decreased breastmilk inflammatory markers, infant fecal microbiome variation, and altered recognition memory responses in infants-a pilot observational study. Front Nutr. (2024) 11:1456111. doi: 10.3389/fnut.2024.1456111, 39385777 PMC11462058

[135] Scher MS Ludington-Hoe S Kaffashi F Johnson MW Holditch-Davis D Loparo KA. Neurophysiologic assessment of brain maturation after an 8-week trial of skin-to-skin contact on preterm infants. Clin Neurophysiol. (2009) 120:1812–8. doi: 10.1016/j.clinph.2009.08.004, 19766056 PMC2928473

[136] Bellizzi S Panu Napodano CM Murgia P. Family-centered care for newborns: a global perspective and review. J Trop Pediatr. (2024) 70:fmae026. doi: 10.1093/tropej/fmae026, 39142805

[137] Charpak N Tessier R Ruiz JG Twenty-Year Follow-up of Kangaroo Mother Care Versus Traditional Care Pediatrics (2017)139:e20162063. 27965377 10.1542/peds.2016-2063

[138] Charpak N Tessier R Ruiz JG Uriza F Hernandez JT Cortes D . Kangaroo mother care had a protective effect on the volume of brain structures in young adults born preterm. Acta Paediatr. (2022) 111:1004–14. doi: 10.1111/apa.16265, 35067976 PMC9303677

[139] Gaudet T. Cultural transformation to a whole health system: lessons learned. Glob Adv Health Med. (2022) 11:2164957x221091452. doi: 10.1177/2164957x221091452, 35478714 PMC9036378

[140] Forrest CB Margolis PA Bailey LC Marsolo K del Beccaro MA Finkelstein JA . PEDSnet: a National Pediatric Learning Health System. J Am Med Inform Assoc. (2014) 21:602–6. doi: 10.1136/amiajnl-2014-002743, 24821737 PMC4078288

[141] Harris E. Scoring higher on brain care tool tied to lower dementia, stroke risk. JAMA. (2024) 331:101. doi: 10.1001/jama.2023.25270 38117508

[142] Duffy TP. The Flexner report--100 years later. Yale J Biol Med. (2011) 84:269–76. 21966046 PMC3178858

[143] Martin AK Green TL McCarthy AL Sowa PM Laakso EL. Allied health transdisciplinary models of care in hospital settings: a scoping review. J Interprof Care. (2023) 37:118–30. doi: 10.1080/13561820.2022.2038552, 35341438

[144] Lawless MT Tieu M Archibald MM Pinero De Plaza MA Kitson AL. From promise to practice: how health researchers understand and promote transdisciplinary collaboration. Qual Health Res. (2024) 359:3–16. doi: 10.1177/10497323241235882, 38485670 PMC11626853

[145] Holman D Salway S Bell A Beach B Adebajo A Ali N . Can intersectionality help with understanding and tackling health inequalities? Perspectives of professional stakeholders. Health Res Policy Syst. (2021) 19:97. doi: 10.1186/s12961-021-00742-w, 34172066 PMC8227357

[146] Marcotte KM Gruppen LD. Competency-based education as curriculum and assessment for integrative learning. Educ Sci. (2022) 12:267. doi: 10.3390/educsci12040267

[147] Klebanoff MA. The collaborative perinatal project: a 50-year retrospective. Paediatr Perinat Epidemiol. (2009) 23:2–8. doi: 10.1111/j.1365-3016.2008.00984.x, 19228308 PMC2646177

[148] Owolabi MO Leonardi M Bassetti C Jaarsma J. Global synergistic actions to improve brain health for human development. Nat Rev Neurol. (2023) 19:371–83. doi: 10.1038/s41582-023-00808-z, 37208496 PMC10197060

[149] Migliore A Tagliaro C Schaumann D Hua Y. "University hubs: hybrid spaces between campus, work, and social spaces". In: Mariotti I Tomaz E Micek G Méndez-Ortega C, editors. Evolution of New Working Spaces: Changing Nature and Geographies. Cham: Springer Nature Switzerland (2024). p. 47–58.

[150] Dahabreh IJ Bibbins-Domingo K. Causal inference about the effects of interventions from observational studies in medical journals. JAMA. (2024) 331:1845–53. doi: 10.1001/jama.2024.7741 38722735

[151] Avery CL Howard AG Ballou AF. Strengthening Causal Inference in Exposomics Research: Application of genetic data and Methods. Environmental Health Perspectives (2022) 130:565001. doi: 10.1289/EHP9098 PMC9084332 35533073

[152] McGinnis J Fineberg HV Dzau VJ. Shared Commitments for Health and Health care: A Trust Framework from the Learning Health system.NAM Perspectives. Commentary (2024). doi: 10.31478/202412c PMC11875543 40034331

[153] Ramey SL Msall ME Ramey CT. Paradoxes in pediatric rehabilitation: building an interdisciplinary, total-child framework to promote effective interventions and life course well-being. Front Pediatr. (2025) 13:1540479. doi: 10.3389/fped.2025.1540479, 40129700 PMC11931064

